# Comparative transcriptome and metabolome analysis suggests bottlenecks that limit seed and oil yields in transgenic *Camelina sativa* expressing diacylglycerol acyltransferase 1 and glycerol-3-phosphate dehydrogenase

**DOI:** 10.1186/s13068-018-1326-2

**Published:** 2018-12-19

**Authors:** Hesham M. Abdullah, Sudesh Chhikara, Parisa Akbari, Danny J. Schnell, Ashwani Pareek, Om Parkash Dhankher

**Affiliations:** 1 Stockbridge School of Agriculture, University of Massachusetts, Amherst, MA 01003 USA; 2 Biotechnology Department, Faculty of Agriculture, Al-Azhar University, Cairo, 11651 Egypt; 3 Department of Plant Biology, Michigan State University, East Lansing, MI 48824 USA; 4 Stress Physiology and Molecular Biology Laboratory, School of Life Sciences, Jawaharlal Nehru University, New Delhi, 100067 India; 5 Present Address: Department of Plant Biology, Michigan State University, East Lansing, MI 48824 USA; 6 Present Address: Centre for Biotechnology, Maharshi Dayanand University, Rohtak, 124001 India

**Keywords:** *Camelina sativa*, TAG biosynthesis, Oilseed, RNA-Seq, Metabolome profiling, Metabolic engineering, Diacylglycerol acyltransferase, Glycerol-3-phosphate dehydrogenase

## Abstract

**Background:**

*Camelina sativa* has attracted much interest as alternative renewable resources for biodiesel, other oil-based industrial products and a source for edible oils. Its unique oil attributes attract research to engineering new varieties of improved oil quantity and quality. The overexpression of enzymes catalyzing the synthesis of the glycerol backbone and the sequential conjugation of fatty acids into this backbone is a promising approach for increasing the levels of triacylglycerol (TAG). In a previous study, we co-expressed the diacylglycerol acyltransferase (DGAT1) and glycerol-3-phosphate dehydrogenase (GPD1), involved in TAG metabolism, in Camelina seeds. Transgenic plants exhibited a higher-percentage seed oil content, a greater seed mass, and overall improved seed and oil yields relative to wild-type plants. To further increase seed oil content in Camelina, we utilized metabolite profiling, in conjunction with transcriptome profiling during seed development to examine potential rate-limiting step(s) in the production of building blocks for TAG biosynthesis.

**Results:**

Transcriptomic analysis revealed approximately 2518 and 3136 transcripts differentially regulated at significant levels in DGAT1 and GPD1 transgenics, respectively. These transcripts were found to be involved in various functional categories, including alternative metabolic routes in fatty acid synthesis, TAG assembly, and TAG degradation. We quantified the relative contents of over 240 metabolites. Our results indicate major metabolic switches in transgenic seeds associated with significant changes in the levels of glycerolipids, amino acids, sugars, and organic acids, especially the TCA cycle and glycolysis intermediates.

**Conclusions:**

From the transcriptomic and metabolomic analysis of DGAT1, GPD1 and DGAT1 + GPD1 expressing lines of *C. sativa*, we conclude that TAG production is limited by (1) utilization of fixed carbon from the source tissues supported by the increase in glycolysis pathway metabolites and decreased transcripts levels of transcription factors controlling fatty acids synthesis; (2) TAG accumulation is limited by the activity of lipases/hydrolases that hydrolyze TAG pool supported by the increase in free fatty acids and monoacylglycerols. This comparative transcriptomics and metabolomics approach is useful in understanding the regulation of TAG biosynthesis, identifying bottlenecks, and the corresponding genes controlling these pathways identified as limitations, for generating Camelina varieties with improved seed and oil yields.

**Electronic supplementary material:**

The online version of this article (10.1186/s13068-018-1326-2) contains supplementary material, which is available to authorized users.

## Background

*Camelina sativa* (L.) Crantz, a member of the Brassicaceae family, has attracted much interest in the recent decades as an emerging oilseed crop as a feedstock for biofuels and industrial chemicals. The agronomic attributes and oil qualities render Camelina an ideal crop for plant breeding programs to improve key traits for food and nonfood purposes. Camelina seed is rich in oil (30–40% of seed dry weight), with a favorable endogenous fatty acid composition as it contains substantially high omega-3 fatty acid (α-linolenic acid—C18:3*n*-3, ALA) content, which is of commercial interests for nutritional values [1, 2]. As an added value to Camelina seed for livestock feed, the seed storage proteins represent an extra 30% of its seed weight, and the seed meal contains relatively lower levels of the toxic glucosinolates as compared to other Brassicaceae species [3, 4]. Further, Camelina can be cultivated on marginal lands, in cold climates, and under drought-like conditions, where other oilseed crops produce relatively lower seed yield [5, 6]. Furthermore, Camelina requires low nutrient inputs and reaches maturity in 90–100 days, so it can be planted as a cover crop in double-cropping systems and thus cultivation/production cost can be reduced [7]. Moreover, a rapid, efficient, and robust genetic transformation via floral dip infiltration method has been developed, which facilitates gene transfer into Camelina for desirable traits [8]. Altogether, Camelina is an ideal candidate for improving agronomic and oil qualities to achieve large-scale and cost-competitive production of renewable biofuels. Consequently, in recent years, Camelina has been subjected to biotechnological improvements to increase seed oil content [4, 9–13], to alter oil composition to better-fit industrial applications [3, 13–22], and to improve the overall seed productivity and plant growth development [4, 10, 12, 23].

In a recent study [12], we overexpressed two enzymes involved in TAG metabolism, the diacylglycerol acyltransferase (DGAT1, EC 2.3.1.20) and glycerol-3-phosphate dehydrogenase (GPD1, EC 1.1.1.8), under the control of seed-specific promoters. We used a transgenic approach to investigate the importance of Gly3P supply for use as the backbone for TAG synthesis, and the importance of acylation with fatty acids in the downstream process for TAG synthesis. Further, we investigated the effect of stacking these two genes in achieving a synergistic effect on the flux through the TAG synthesis pathway, and thereby further increase the oil yield. The transgenic Camelina plants exhibited up to 13% higher seed oil content and up to 52% increase in seed mass, with a great impact on seed and oil yields and significant major switches in fatty acid content and composition, compared to wild-type plants [12].

Although, a previous study [24] unveiled major changes in transcripts and hormonal profiles of transgenic Arabidopsis overexpressing DGAT1, no reports of the effect of GPD1 in transcript and metabolite networks have been published. Additionally, to our knowledge, there is only one report, which addressed the metabolome profiling of *C. sativa* during seed development [25]. Therefore, our data reported here complement and extend the previous studies by providing a broad overview of changes in transcripts and metabolite profiles in transgenic Camelina lines overexpressing *DGAT1* in combination with *GPD1* genes.

Given that very few transcriptome and metabolome profiling studies have been reported in Camelina, we are interested in exploiting transgenic Camelina plants exhibiting improved seed and oil yields to expand our understanding of TAG biosynthesis and determine the molecular and biochemical consequences of pushing the seed and oil production pathways forward. In this study, we performed transcript and metabolite profiling of transgenic *C. sativa* overexpressing *DGAT1* and *GPD1* genes, individually or combined, at several different seed developmental stages. The integration of transcriptome and metabolome is highly useful for understanding the regulation of TAG biosynthesis and identifying the bottlenecks toward metabolic engineering of Camelina varieties with improved seed and oil qualities.

## Results and discussion

### Global changes in seed transcriptome associated with overexpression of AtDGAT1 and ScGPD1

In the current study, we analyzed transgenic *C. sativa* (*cv.* Suneson) lines overexpressing the Arabidopsis *DGAT1* (*AtDGAT1*), driven by the seed-specific glycinin promoter (DGAT1 line #2), or *Saccharomyces* *cerevisiae GPD1* (*ScGPD1*), driven by the seed-specific oleosin promoter (GPD1 line # 2), or the combined line co-expressing *AtDGAT1* and *ScGPD1* (GPD1 + DGAT1 line #11). These lines were selected for this study because they accumulated substantially higher seed oil content, produced larger seeds, and produced relatively higher seed and oil yields than the non-transgenic WT control. Detailed molecular, biochemical, phenotypic, and physiological characterizations of these three lines along with other comparable lines of Camelina were published previously [12].

Illumina sequencing was performed on cDNA libraries prepared from Camelina seeds at 10–15 and 16–21 days after flowering (DAF) in the homozygous T3 generation of DGAT1 #2 and GPD1 #2 lines to address the changes in gene expressions during seed development compared to non-transgenic WT seeds. Paired-end 100-base sequencing generated between 36 and 97 million reads per library using three biological replicates. Reads were aligned to the Camelina reference genome, and the mRNA expression levels for Camelina genes were assessed. Overall, over 96% of the reads were successfully aligned to the reference genome, regardless of the genotype analyzed or the seed developmental stage (Additional file 1: Table S1).

**Fig. 1:**
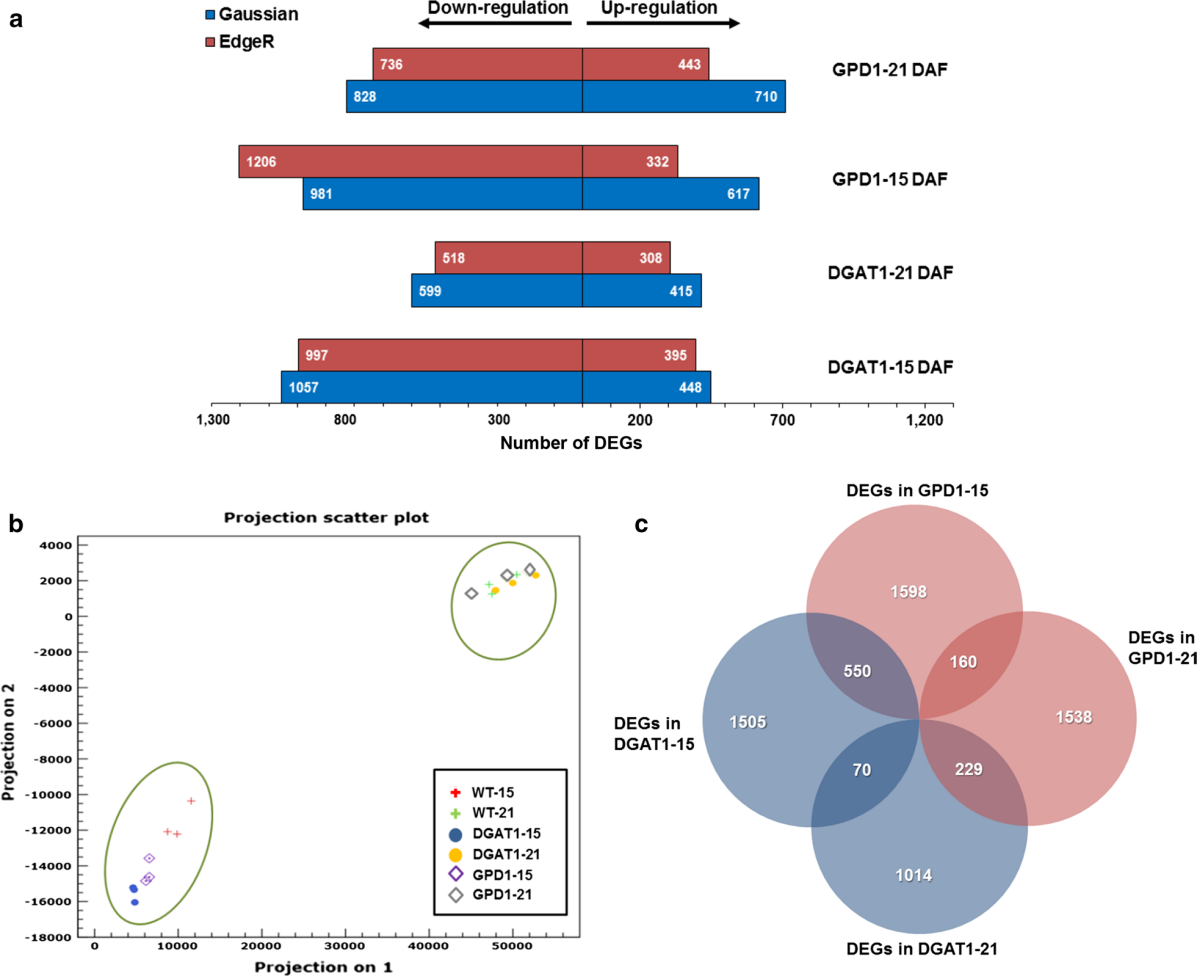
Global changes in the transcriptome profiles in Camelina transgenic lines and wild-type developing seeds. **a** The number of DEGs and the regulation in DGAT1 and GPD1 lines relative to that in WT is summarized. **b** Principal component analysis (PCA) indicates the variability of RNA-Seq datasets between WT and transgenic lines in the indicated time points after flowering, and **c** Venn diagram showing the overlapped relationships between DEGs in DGAT1 and GPD1 lines as compared to WT data. DEGs, differentially expressed genes, WT-15, GPD1–15, and DGAT1–15 indicate the wild-type and transgenic lines data of developing seeds harvested at 10–15 DAF, whereas WT-21, GPD1–21, and DGAT1–21 indicate the wild-type and transgenic lines data of developing seeds harvested at 16–21 DAF. Gaussian and EdgeR indicate the two pipelines analysis platforms used to determine the DEGs. DAF, days after flowering. WT, wild-type; GPD1, lines overexpressing *ScGPD1* gene; and DGAT1, lines overexpressing *AtDGAT1* gene

For accurate identification of the differentially expressed genes (DEGs) and estimation of their expression patterns, we analyzed the RNA-Seq data using the two methods EdgeR and Gaussian tests [26] (CLC Genomics Workbench 8.0.3, https://www.qiagenbioinformatics.com). To get a global view on the transcriptomic changes that occur during seed development, the RNA-Seq data were statistically analyzed, and the results were presented in multiple ways (Fig. 1, also see the volcano plots in Additional file 1: Figs. S1, S2). The principal component analysis (PCA) indicated that the RNA-Seq datasets from control and transgenic lines showed less variation within a developmental stage than a comparison of the same genotype between different developmental stages. However, the sample variation was the highest between WT and both DGAT1 and GPD1 lines at early seed stages (10–15 DAF, Fig. 1b).

To identify the genes that are differentially expressed between Camelina transgenics and WT, we compared the transcript levels of Camelina genes in the two seed stages (10–15 and 16–21 DAF). The DEGs were highlighted (Fig. 1), which showed ≥ 1.5-fold expression changes (*P* value ≤ 0.05) and were confirmed to be actively expressed (RPKM ≥ 0.1, in log_2_ scale). The significance analysis revealed variations in the DEGs identified using the two methods applied in the current study. Overall, more genes were identified as being down-regulated rather than up-regulated in Camelina transgenics compared to the WT control. The EdgeR-based analysis identified a total of 2218 and 2717 DEGs in DGAT1 and GPD1 lines, respectively, compared to WT during the two indicated stages of seed development. Of these, expression of 703 and 1515 genes was up and down-regulated, respectively, in the DGAT1 line, while expression of 775 and 1942 genes was up- and down-regulated, respectively, in the GPD1 line (Fig. 1a).

On the other hand, the Gaussian analysis identified a total of 2519 and 3136 DEGs in DGAT1 and GPD1 lines, respectively, compared to WT during the two indicated stages of seed development. A total of 863 transcripts were up-regulated and 1656 were down-regulated in the DGAT1 line, and 1327 transcripts were up-regulated and 1809 down-regulated in the GPD1 line (Fig. 1a). The difference in the numbers of DEGs identified by both EdgeR and Gaussian analysis methods could be associated with the variation of the analysis parameters used and the mapping approaches used in the two methods.

Furthermore, 550 and 229 DEGs in 10–15 and 16–21 DAF samples, respectively, were common to both DGAT1 and GPD1 seeds (Fig. 1c). However, only 70 DEGs in DGAT1 and 160 DEGs in GPD1 were common to both seed stages (Fig. 1c). This observation indicated that DGAT1 and GPD1 expression in Camelina seeds affects certain common metabolic pathways during seed development. A full list of the DEGs in DGAT1 and GPD1 transgenic lines, relative to WT, in two seed developmental stages, is provided in Additional file 2: Table S2, DGAT1 vs. WT (10–15 DAF), DGAT1 vs. WT (16–21 DAF), GPD1 vs. WT (10–15 DAF), and GPD1 vs. WT (16–21 DAF).

### Annotation and gene ontology (GO) of the DEGs

The genomes of Camelina and its close relatives, Arabidopsis and Brassica, are fully sequenced (http://www.camelinadb.ca, *Cs_genome_sequence_build_V2.0*, http://www.arabidopsis.org, and http://www.brassica.info, respectively). Therefore, we relied on the information of the gene ontology (GO) annotation obtained from these genomes to identify the functional classifications of the DEGs in Camelina transgenics relative to WT. Overall, the GO enrichment analysis of the DEGs has indicated that the DEGs encode proteins involved in various molecular functions, and controlling different metabolic pathways (Table 1 and Additional file 3: Table S3, Additional file 4: Table S4, Additional file 5: Table S5, Additional file 6: Table S6, Additional file 7: Table S7, Additional file 8: Table S8, Additional file 9: Table S9, Additional file 10: Table S10). The GO classification shown in Table 1 contains the predicted molecular function of the DEGs in Camelina transgenic lines analyzed in the current study. During Camelina seed development, the overexpression of DGAT1 or GPD1 was shown to cause significant changes in the expression of a large group of genes belonging to lipid binding, catalytic, hydrolase, and transferase activities (Table 1).

**Table 1:** Functional classifications of the DEGs in Camelina transgenics as compared to WT

GO ID	GO term	Counts							
		Up in DG-14	Down in DG-14	Up in DG-21	Down in DG-21	Up in GP-14	Down in GP-14	Up in GP-21	Down in GP-21
GO:0005488	Binding	80	236	100	152	159	198	178	211
GO:0003824	Catalytic activity	68	282	85	121	130	200	158	173
GO:1901363	Heterocyclic compound binding	24	145	57	91	93	108	116	126
GO:0016787	Hydrolase activity	21	89	30	31	42	57	61	49
GO:0016788	Hydrolase activity, acting on ester bonds	4	29	11	8	13	23	20	12
GO:0043167	Ion binding	52	149	54	87	92	119	104	84
GO:0016874	Ligase activity	4	10	2	3	2	10	1	7
GO:0008289	Lipid binding	4	8	4	1	3	8	7	4
GO:0003676	Nucleic acid binding	24	54	27	55	49	50	48	59
GO:0001071	Nucleic acid binding transcription factor activity	14	29	8	20	24	28	18	30
GO:0097159	Organic cyclic compound binding	24	145	57	91	93	108	116	126
GO:0016491	Oxidoreductase activity	16	81	15	35	31	59	31	42
GO:0005515	Protein binding	2	37	20	20	24	37	34	47
GO:0016740	Transferase activity	28	86	35	50	55	56	70	70
GO:0016746	Transferase activity, transferring acyl groups	4	15	6	6	9	9	9	12
GO:0005215	Transporter activity	12	28	14	10	18	20	22	13
GO:0016209	Antioxidant activity	–	12	–	1	2	8	–	–
GO:0030246	Carbohydrate binding	–	6	2	6	4	5	1	6
GO:0097367	Carbohydrate derivative binding	–	46	21	22	30	31	44	31
GO:0050662	Coenzyme binding	–	5	4	5	2	5	7	9
GO:0048037	Cofactor binding	–	7	6	6	5	7	9	11
GO:0009055	Electron carrier activity	–	5	1	4	3	4	–	3
GO:0016301	Kinase activity	13	27	13	12	16	17	20	18
GO:0016829	Lyase activity	1	19	2	3	3	14	6	2
GO:0046872	Metal ion binding	–	98	30	59	60	88	61	82
GO:0045735	Nutrient reservoir activity	–	22	–	1	–	17	1	1
GO:0000166	Nucleotide binding	–	53	26	31	36	41	58	43
GO:0019825	Oxygen binding	–	14	–	2	3	8	5	3
GO:0005524	ATP binding	–	44	20	19	25	27	34	28
GO:0016887	ATPase activity	7	14	4	4	7	4	9	5
GO:0008233	Peptidase activity	5	17	6	7	5	8	6	10
GO:0042578	Phosphoric ester hydrolase activity	2	9	4	4	7	12	7	5
GO:0016157	Sucrose synthase activity	–	1	–	–	–	1	–	–
GO:0046524	Sucrose-phosphate synthase activity	–	1	–	–	–	–	–	–
GO:0016298	Lipase activity	–	6	2	–	2	3	3	1
GO:0004806	Triglyceride lipase activity	–	–	2	–	1	–	1	–
GO:0005319	Lipid transporter activity	–	1	1	–	–	–	–	–
GO:0047617	Acyl-CoA hydrolase activity	–	–	–	–	–	–	–	1
GO:0016411	Acylglycerol *O*-acyltransferase activity	–	1	–	1	–	–	3	–
GO:0016421	CoA carboxylase activity	–	–	–	–	–	–		1
GO:0047734	CDP-glycerol diphosphatase activity	–	–	1	–	–	–	–	–
GO:0004144	Diacylglycerol *O*-acyltransferase activity	–	1	–	1	–	–	2	–
GO:0090447	Glycerol-3-phosphate 2-*O*-acyltransferase activity	–	–	–	–	–	–	1	1
GO:0004366	Glycerol-3-phosphate *O*-acyltransferase activity	–	–	–	–	–	–	1	1
GO:0052722	Fatty acid in-chain hydroxylase activity	–	1	–	–	–	–	–	–
GO:0080019	Fatty-acyl-CoA reductase (alcohol-forming) activity	–	1	–	–	–	–	–	–
GO:0015254	Glycerol channel activity	–	3	–	–	–	3	–	–
GO:0015168	Glycerol transmembrane transporter activity	–	3	–	–	–	4	–	–
GO:0090447	Glycerol-3-phosphate 2-*O*-acyltransferase activity	–	3	1	–	–	2	–	–
GO:0004366	Glycerol-3-phosphate *O*-acyltransferase activity	–	–	1	–	–	–	–	–
GO:0050062	Long-chain-fatty-acyl-CoA reductase activity	–	2	–	–	–	1	–	–
GO:0042171	Lysophosphatidic acid acyltransferase activity	–	–	–	–	–	–	1	–
GO:0071617	Lysophospholipid acyltransferase activity	–	–	–	–	–	–	1	–
GO:0008374	*O*-Acyltransferase activity	–	4	2	1	1	2	4	1
GO:0004607	Phosphatidylcholine-sterol *O*-acyltransferase activity	–	–	–	–	1	–	–	–
GO:0004623	Phospholipase A2 activity	–	–	–	–	1	–	–	–
GO:0008429	Phosphatidylethanolamine binding	–	3	–	–	–	2	–	1
GO:0004435	Phosphatidylinositol phospholipase C activity	–	1	–	–	–	–	–	–
GO:0004620	Phospholipase activity	–	1	–	–	1	1	–	1
GO:0004629	Phospholipase C activity	–	1	–	–	–	1	–	–
GO:0035091	Phosphatidylinositol binding	–	–	1	–	–	–	1	–
GO:0005543	Phospholipid binding	–	3	1	1	1	2	1	–
GO:0005548	Phospholipid transporter activity	–	–	1	–	–	–	–	–
GO:0004012	Phospholipid-translocating ATPase activity	–	–	1	–	–	–	–	–
GO:0016412	Serine *O*-acyltransferase activity	–	–	1	–	–	–	–	–

Shown is the gene ontology (GO) annotation of the selected differentially expressed genes (DEGs) in Camelina transgenic lines overexpressing DGAT1 or GPD1 in developing seeds at 10–15 and 16–21 days after flowering (DAF). The GO ID, description and DEGs numbers are provided. Up in DG-14 or DG-21 indicate the genes up-regulated in DGAT1 #2 line in developing seeds at 10–15 and 16–21, respectively; Down in DG-14 or DG-21 indicate the genes downregulated in DGAT1 #2 line in developing seeds at 10–15 and 16–21, respectively; Up in GP-14 or GP-21 indicate the genes up-regulated in GPD1 #2 line in developing seeds at 10–15 and 16–21, respectively; Down in GP-14 or GP-21 indicate the genes downregulated in GPD1 #2 line in developing seeds at 10–15 and 16–21, respectively

Notably, a large number of DEGs were identified to encode proteins that can bind to ions (342 in DGAT1 and 399 in GPD1), lipids (17 in DGAT1 and 22 in GPD1), proteins (79 in DGAT1 and 142 in GPD1), nucleotides (110 in DGAT1 and 178 in GPD1), carbohydrate derivatives (89 in DGAT1 and 136 in GPD1), transcription factors (71 in DGAT1 and 100 in GPD1), and ATP (83 in DGAT1 and 114 in GPD1). Further, many of the DEGs were associated with either hydrolase or transferase activities, and a total of 171 and 209 hydrolases and a total of 199 and 251 transferases were developmentally regulated in DGAT1 and GPD1 lines, respectively. Among these hydrolases, many were found to act on ester bonds, and among transferases, many can transfer acyl groups. Considering a 1.5-fold-change cut-off of the genes identified to be differentially expressed (*P* value ≤ 0.05), we highlighted the genes showing the highest levels of expression which are either up-regulated or down-regulated in response to DGAT1 or GPD1 overexpression (Additional file 1: Tables S11, S12). As shown in the tables, many genes were shown to be up-regulated in Camelina seeds in response to the overexpression of DGAT1. Those included genes involved in lipid transport, genes belonging to the gibberellin-regulated family, which play a role in plant development [27], plant defensins (shown in Additional file 1: Table S11 as defensin 46, isoflavone reductase homolog P3-like, and Kunitz-type serine protease inhibitor-like), which have no confirmed roles in lipid metabolism, but are active as antibacterials and antifungals during embryo development [28]. Also, a group of seed-specific genes involved in preparing seeds for germination (shown as proline-rich extensin EPR1) were also up-regulated. Further, multiple lipid transfer proteins (LTPs) were also identified among the genes that were up-regulated in DGAT1 transgenics. LTPs play a critical role in in vitro transfer of phospholipids across membranes and regulate intracellular fatty acid pools, as reported previously [24, 29].

Furthermore, the list of DEGs also contained various genes encoding seed storage proteins and oleosins, which were down-regulated in DGAT1 transgenics. Genes encoding seed storage proteins cruciferin 3 and 2S albumin, and the oil body membrane proteins oleosin 5 and oleosin 2 were dominant among the DEGs whose expression was negatively affected by DGAT1 overexpression in Camelina seeds. It was reported that Oleosin 5, in particular, was shown to be involved in stabilizing the lipid body during seed desiccation, thus preventing coalescence of the oil [30]. It probably interacts with both lipid and phospholipid moieties of lipid bodies, and may also provide recognition signals for specific lipases to act in lipolysis during seed germination and post-germinative growth [31].

Additionally, the annotation analysis for the DEGs in GPD1 transgenic seeds revealed similar transcriptional effects as in DGAT1 transgenic seeds. Genes encoding gibberellin-regulated proteins, desiccation and oxidative stress-associated proteins (plant defensins, isoflavone reductases, and 5-adenylylsulfate reductases), and senescence-associated proteins (i.e., tropinone reductases) were up-regulated in GPD1 seeds. Comparable to DGAT1 lines, overexpression of GPD1 in Camelina seeds was associated with down-regulation of several genes encoding seed storage proteins and oleosins, genes encoding proteins involved in promoting cell elongation and organ growth (glycine-rich cell wall structural-like), and genes involved in photosynthesis, particularly light harvesting in photosystems I and II, in response to seed maturation (see Additional file 1: Table S12).

Since overexpression of DGAT1 and/or GPD1 enzymes had positively impacted the seed and oil production in Camelina as reported in our previous study [12], here we highlighted the DEGs with lipid-related functions or that are key regulators of many seed processes, including seed maturation and oil accumulation. 89 and 90 transcripts implicated in lipid-related functions were differentially expressed in DGAT1 and GPD1 lines, respectively. 37 transcripts were up-regulated and 52 transcripts were down-regulated in DGAT1 lines, while a total of 55 transcripts were up-regulated and 35 transcripts were down-regulated in GPD1 lines (Additional file 1: Table S13). The overexpression of DGAT1 resulted in up-regulation of transcripts encoding enzymes involved in fatty acid synthesis, including 3-ketoacyl-CoA synthase 2, which is required for fatty acid elongation and storage in developing seeds [32], and a pyruvate kinase, which synthesizes pyruvate from d-glyceraldehyde 3-phosphate and plays a role in seed oil accumulation and embryo development [33]. Further, the expression of genes encoding enzymes of the Kennedy pathway of TAG synthesis; glycerol-3-phosphate acyltransferase 4 (GPAT4) and lysophosphatidyl acyltransferase 4 (LPAT4), or those utilizing membrane-localized phospholipids; phosphatidic acid phosphatase (PAP2) and non-specific phospholipase C4 (NPC4), to supply diacylglycerols (DAGs) was shown to be elevated in DGAT1 lines. Since many of the DEGs in DGAT1 lines were shown to be involved in lipid synthesis, transport, and storage, these findings are consistent with the previous report [24], suggesting the critical impact of DGAT1 overexpression on those processes. Nevertheless, none of these lipid-related genes have been characterized in Camelina.

On the other hand, overexpression of GPD1 caused up-regulation of the genes encoding enzymes involved in fatty acid synthesis (i.e., pyruvate kinase), transfer (i.e., LTP4 and LTP6), and activation (i.e., acyl-activating enzyme 17), in addition to the genes encoding enzymes involved in TAG biosynthetic pathways such as glycerol-3-phosphate acyltransferase 1 (GPAT1), lysophosphatidyl acyltransferase 5 (LPAT5), *O*-acyltransferase (WSD1-like), and phospholipases (i.e., phospholipase A2-beta, and phospholipase C1; Additional file 1: Table S13).

Due to the critical roles of transcriptional regulation of diverse biological processes, including seed development and oil accumulation, we were curious to investigate whether the overexpression of DGAT1 and/or GPD1 in Camelina seeds had impacted the expression levels of transcription factors (TFs). Since many transcription factors were reported to govern the expression of multiple enzymes in the oil metabolic pathways, and many are critical for seed development and overall plant growth [34, 35], any changes in the TFs transcriptional activity could contribute to desired changes in seed and/or oil yields in Camelina [9, 36], or alternatively lead to unwanted side effects [37]. In this regard, we highlighted the DEGs encoding TFs that are shown to be differentially regulated in response to the overexpression of DGAT1 or GPD1 in transgenic Camelina, relative to the WT plants (Additional file 1: Table S14). The analysis of the DEGs identified a total of 16 and 47 genes that were up-regulated and down-regulated in DGAT1 line, respectively, while a total of 28 and 45 genes were up-regulated and down-regulated in GPD1 line, respectively. The GO annotation for those identified genes indicated that none of the transcription factors that were previously identified as key regulators for oil accumulation in seeds [38–41] were present in the DEGs list in DGAT1 and GPD1 lines. But, many transcription factors regulating non-lipid-specific functions were also observed in the DEGs list, for instance, the genes encode (i) the ethylene-responsive (ERF) TFs, which regulate plant development and tolerance to abiotic stresses [42], (ii) DNA-binding One Zinc Finger (DOF) TFs, which have roles in seed maturation and germination [43], (iii) WRKY TFs, which show diverse functions, including seed development, senescence, nutrient deprivation, and abiotic stress responses [44], and (iv) NAC domain-containing TFs, which regulate auxin signaling in lateral root development [45].

### Validation of transcript abundance using qRT‑PCR

**Fig. 2:**
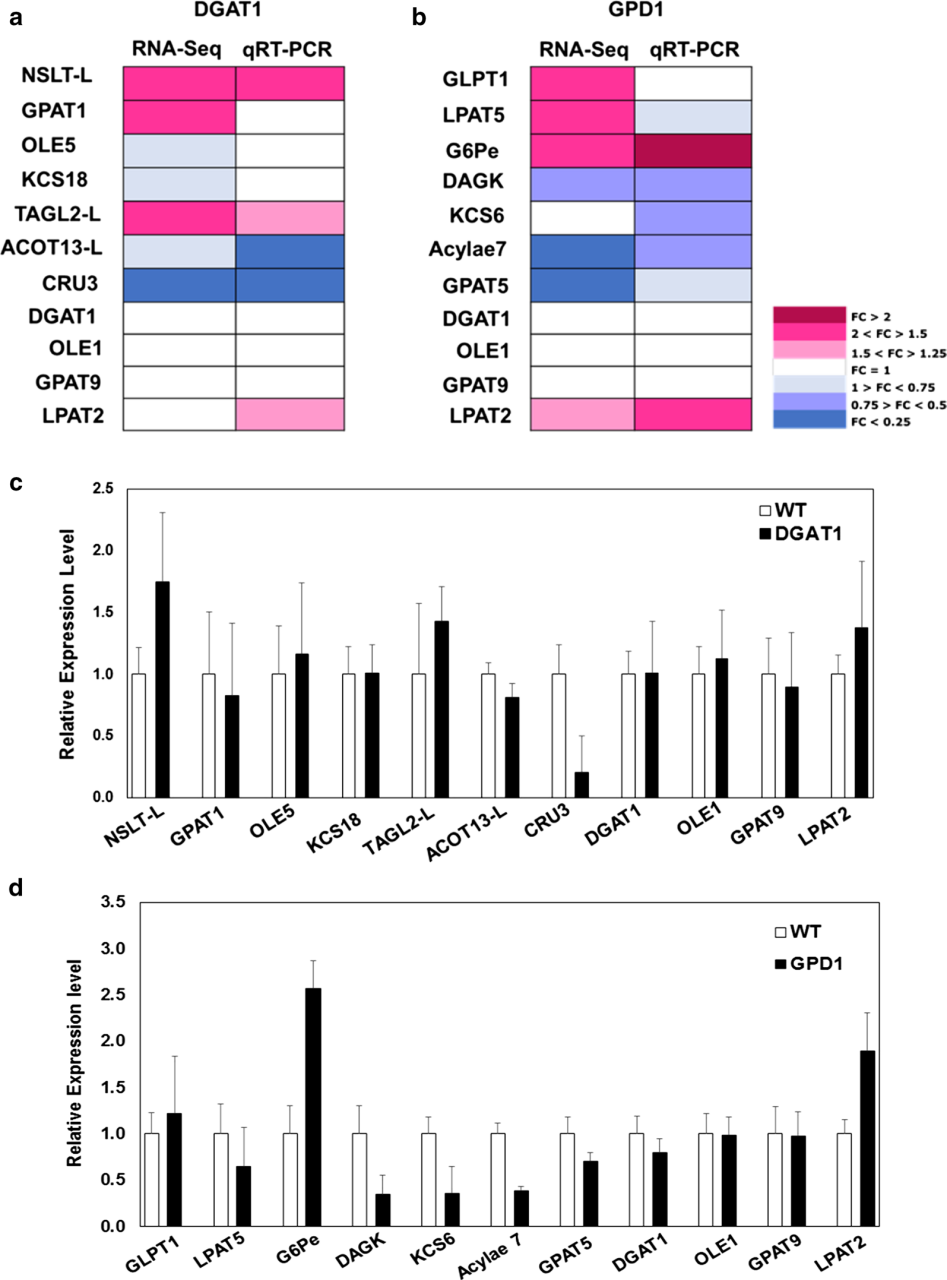
The gene expression analysis for the selected genes showing differential regulation in Camelina transgenic lines. Data are the fold changes (FC) in expression measured by using both RNA-Seq and qRT-PCR techniques (**a**, **b**) in both DGAT1 and GPD1, respectively, relative to WT. The fold change values used in the analysis are presented in Additional file 1: Table S15. Data shown in **c**, **d** indicate the relative gene expression for the selected genes measured by qRT-PCR in both DGAT1 and GPD1 lines, respectively, relative to WT. The genes shown here are non-specific lipid transfer 4-like (*NSLT*-*L*), glycerol-3-phosphate *sn*-2-acyltransferase 1 (*GPAT1*), oleosin 5 (*OLE5*), 3-ketoacyl-synthase 18-like (*KCS18*), TAG-lipase 2-like (*TAGL2*-*L*), acyl CoA thioesterase 13-like (*ACOT13*-*L*), cruciferin 3 (*CRU3*), acyl-CoA:diacylglycerol acyltransferase 1 (*DGAT1*), oleosin 1 (*OLE1*), glycerol-3-phosphate acyltransferase 9 (*GPAT9*), lysophosphatidyl acyltransferase 2 (*LPAT2*), glycerol-3-phosphate transporter 1 (*GLPT1*), lysophosphatidyl acyltransferase 5 (*LPAT5*), glucose-6-phosphate l-epimerase (*G6Pe*), diacylglycerol kinase 3-like isoform X1 (*DAGK*), 3-keto acyl-synthase 6 (*KCS6*), acyl-activating enzyme 7 (*Acylae7*), glycerol-3-phosphate acyltransferase 5 (*GPAT5*)

To verify the RNA-Seq results, the relative gene expression of a total of selected 17 candidate genes was measured by qRT-PCR, using RNA templates obtained from developing seeds at 16–21 DAF (Fig. 2 and Additional file 1: Table S15). The listed genes were selected for the current analysis due to the roles they play in lipid metabolism in seeds as previously reported and the differential gene expression levels they exhibited during Camelina seed development. As shown in Fig. 2, we reported the genes to be up-regulated, if we observed a fold change (FC) > 1.25, or down-regulated if FC < 0.75, or unchanged if 1.25 > FC < 0.75, in Camelina transgenics relative to WT.

Among the 11 genes tested in DGAT1 lines, 5 genes showed similar expression patterns when tested by both qPCR and RNA-Seq techniques. The qPCR analysis indicated that overexpression of DGAT1 has no impact on the genes involved in TAG assembly and accumulation, GPAT9, OLE1, and the indigenous DGAT1, but caused significant up-regulation of the genes encoding the Non-specific lipid transfer 4-like (NSLT-L), which play vital roles in seed development and germination [46], and the TAG lipase (TAGL2-L), which catalyzes the hydrolysis of TAGs to form glycerol and fatty acids [47]. Whereas, DGAT1 overexpression significantly caused down-regulation of the gene encoding the seed storage protein Cruciferin 3, CRU3 (Fig. 2 and Additional file 1: Table S15).

On the other hand, there was a stronger agreement in the expression levels measured by qPCR and RNA-Seq in GPD1 transgenic lines, relative to WT. The qRT-PCR verified the expression levels of 8 out of 11 genes tested in GPD1 lines and the results were consistent with RNA-Seq results (Fig. 2 and Additional file 1: Table S15). Of those, 2 genes were significantly up-regulated, 4 genes were down-regulated, while 3 genes observed no changes, in response to GPD1 overexpression in Camelina transgenics. The overexpression of GPD1 in Camelina seeds led to a significant increase in the expression levels of the genes encoding glucose-6-phosphate l-epimerase (G6Pe), an enzyme participating in glycolysis/gluconeogenesis in *S. cerevisiae*, [48], and the gene encoding lysophosphatidyl acyltransferase 2 (LPAT2), an endoplasmic reticulum-located protein involved in the conversion of lysophosphatidic acid (LPA) into phosphatidic acid (PA) by incorporating an acyl moiety at the *sn*-2 position, a critical step in TAG assembly [49]. Further, the qRT-PCR analysis indicated that the expression of *GPD1* gene has caused a significant reduction in the expression levels of a few genes involved in fatty acid synthesis and activation in Camelina seeds. A significant reduction in gene expression was detected for a gene encoding a member of 3-ketoacyl-CoA synthase family (namely, KCS6), which is required for the synthesis of very long-chain fatty acids (VLCFAs, [50]), a gene encoding a member of acyl-activating enzymes family with diverse biological functions among plant species [51], a gene encoding a protein with acyl-CoA:glycerol-3-phosphate acyltransferase activity (GPAT5), which have no roles in seed TAG accumulation, but plays a critical role in polyester biogenesis in seed coats and roots [52], and a gene encoding a member of diacylglycerol kinases (DAGK), which catalyze the conversion of DAG into phosphatidic acid (PA), and thus implicated in signal transduction pathways in plants [53]. Moreover, similar to the case in DGAT1 lines, GPD1 expression causes no change in the expression of TAG assembly-related genes (i.e., OLE1, DGAT1, and GPAT9) as presented in Fig. 2 and Additional file 1: Table S15.

The reasons why the expression levels detected for some genes measured by qRT-PCR do not correlate with the expression levels detected in the RNA-Seq analysis could be due to the polyploidy nature of Camelina genome and the technical parameters applied in both techniques. Camelina has a hexaploid genome structure where there are three closely related expressed subgenomes and each gene in *A. thaliana* was shown to match with the corresponding triplicates of *C. sativa* homologs as Camelina genes were found to be syntenically orthologous to Arabidopsis genes [54]. The polyploidy of the Camelina genome raised a challenge to detect the expression of a single gene copy using the accessible and limited routines included in the RNA-Seq data analysis. To validate the gene expression in the current study using qRT-PCR, we needed to design the PCR primers to target a conserved sequence region of the three gene copies, and as a result, the gene expression reported is the aggregate expression for the triplicates.

The full names of the selected genes and more details on their expression levels detected by either qPCR or RNA-Seq analysis as well as the PCR primers used to measure gene expression are available in Additional file 1: Tables S15, S16.

### Overexpressing AtDGAT1 and/or ScGPD1 causes global switches in Camelina metabolite profiles

The dataset of metabolome profiles presented in this study comprises a total of 246 compounds of known identity measured by a combination of GC/MS and LC/MS platforms following the analysis pipelines described in “Methods” section. ANOVA contrasts were used to identify biochemicals that differed significantly (*P* < 0.05) between WT and GPD1, DGAT1, or DGAT1 + GPD1 lines in Camelina seeds during development. The detailed information of metabolite contents of Camelina genotypes analyzed is presented as integrated peak raw ion counts, after normalization and log transformation (Additional files 11: Table S17). To understand the effects of expressing the DGAT1 and GPD1 enzymes in developing seeds on metabolites, statistical comparisons of relative metabolite contents in WT and transgenic seeds were performed. The detailed information of relative metabolite ratios and statistical analysis are presented in Additional files 12: Table S18.

**Fig. 3:**
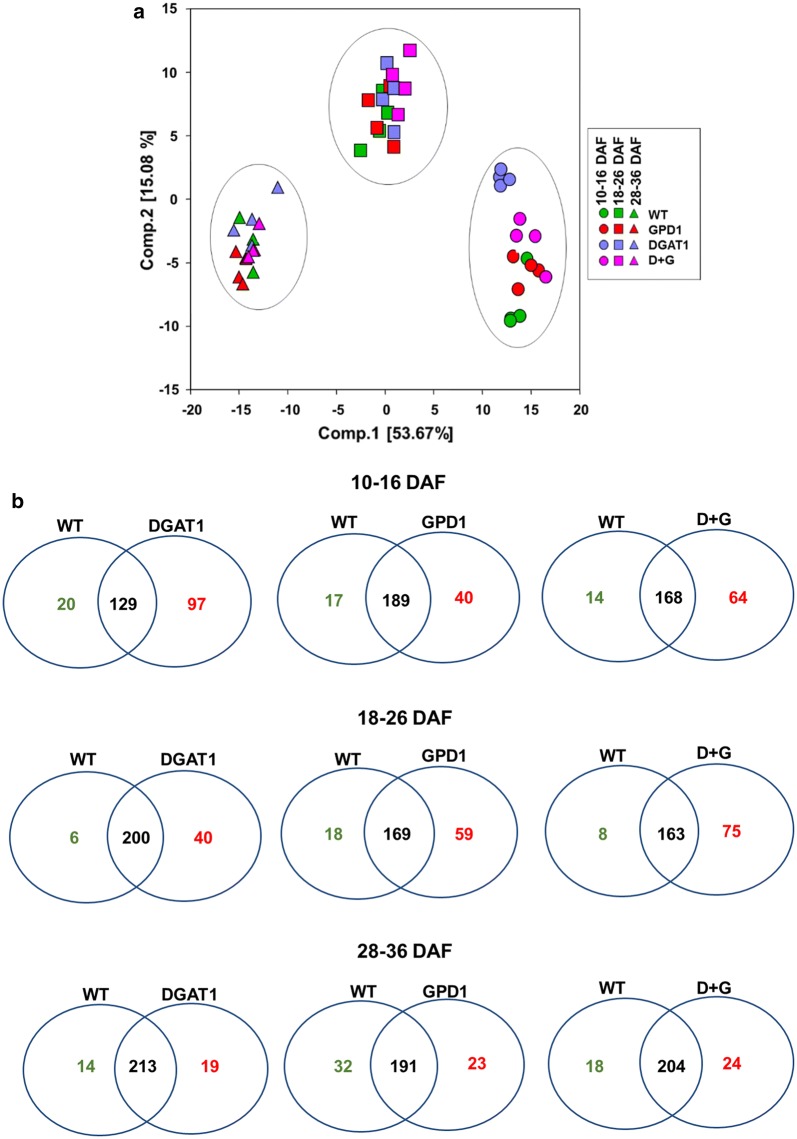
Global changes in the metabolite profiles in Camelina transgenics and WT during the seed development. **a** Principle components analysis (PCA) showing the variability of metabolites contents of Camelina wild-type (WT) and transgenic GPD1, DGAT1, and D + G lines overexpressing both *ScGPD1* and *AtDGAT1* genes. The data represent the variance between the four biological replicates tested in each genotype. **b** Venn diagrams of the global changes of the metabolites abundance between Camelina WT and transgenic lines at three stages of seed development. The number of metabolites showed significant increase in transgenics compared to WT is highlighted in red, number of metabolites showed significant decrease in transgenics compared to WT is highlighted in green, and metabolites with no change are highlighted in black. DAF, days after flowering. WT, wild-type, GPD1, lines overexpressing *ScGPD1* gene, DGAT1, lines overexpressing *AtDGAT1* gene, and D + G, lines overexpressing both *ScGPD1* and *AtDGAT1* genes

We addressed the effects of seed developmental stages (10–16, 18–26, 28–36 DAF) within each genotype as well as the effects of the three transgenic constructs relative to WT within each of the three seed stages. The principal component analysis (PCA) indicated that there was a strong separation between the two developmental stages analyzed, but there was a noticeable separation between genotypes only during the earliest seed stage (10–16 DAF) (Fig. 3a). We also summarized the number of metabolites that are differentially accumulated between WT and transgenic lines in the Venn diagram analysis (Fig. 3b). The two-way ANOVA analysis revealed that there are almost twice as many metabolites altered by the developmental stage compared to the genotype effect. And that, more than half of the metabolites were significantly altered in each seed stage comparisons (relative to stage 1, 10–16 DAF) or in each genotype (relative to the WT). The statistical comparisons of metabolite contents showed that seed stages 1 and 2 (10–16 and 18–26 DAF, respectively) tended to have more alterations than in seed stage 3 (28–36 DAF) and that the DGAT1 line, followed by the DGAT1 + GPD1 line, in stage 1 showed the greatest differences.

**Fig. 4:**
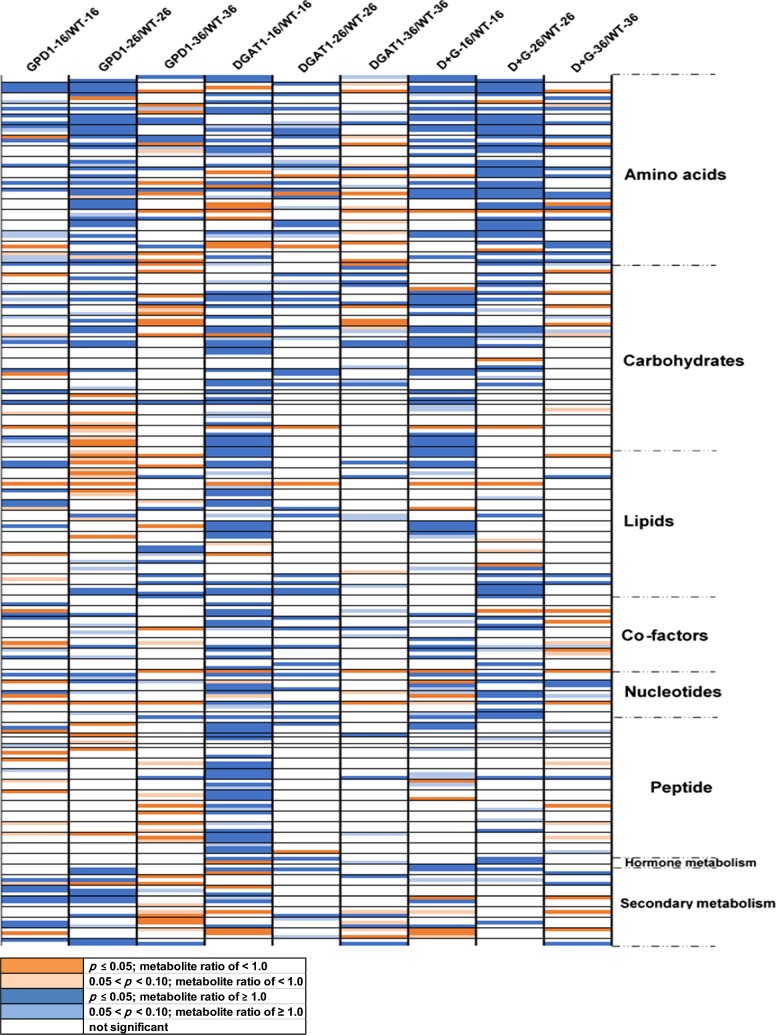
Heat map analysis showing changes in the contents of metabolites during Camelina seed development. The data represent the metabolite content ratios in Camelina transgenic DGAT1, GPD1, and D + G lines relative to WT at 10–16 DAF, 18–26 DAF, and 28–36 DAF. WT data was used as controls in pairwise comparisons. Metabolites showed a ratio of < 1.00 and significant difference (*P* ≤ 0.05) are highlighted in orange, metabolites narrowly missed statistical cutoff for significance 0.05 < *P* < 0.10 and metabolite ratio of < 1.00 are highlighted in light orange, metabolites showed a ratio of ≥ 1.00 and significant difference (*P* ≤ 0.05) are highlighted in blue, metabolites narrowly missed statistical cutoff for significance 0.05 < *P* < 0.10 and metabolite ratio of ≥ 1.00 are highlighted in light blue, and non-colored text and cell means values are not significantly different for that comparison. Values are representative of four biological replicates of developing seeds bulked from at least 8 plants for each time point. The genotypes used are WT, wildtype, DGAT1, AtDGAT1 overexpressor, GPD1, ScGPD1 overexpressor, and D + G, GPD1 + DGAT1 overexpressor. Developing seeds were harvested at 10–16 DAF (WT-16, GPD1–16, DGAT1–16, D + G-16), 18–26 DAF (WT-26, GPD1–26, DGAT1–26, D + G-26), 28–36 DAF (WT-36, GPD1–36, DGAT1–36, D + G-36)

Furthermore, the heat map for the fold change increases or decreases in the relative metabolite contents agreed with results from the PCA and the Venn diagram analyses, that the greatest effect on the data is derived from the developmental stages of seeds (Fig. 4). Relative to WT, we observed higher levels of amino acids, fatty acids, and certain carbohydrates in the early seed stages, particularly in DGAT1 and DGAT1 + GPD1 lines, but their relative levels were significantly lower in later seed stages. Also, the expression of GPD1 was associated with a noticeable increase in the levels of amino acids and secondary metabolites, and a reduction in the levels of certain lipids. This is presumably because of the incorporation of these compounds into proteins and complex lipids.

Furthermore, it is noteworthy to mention that some metabolic effects clearly seemed to be isolated to one of the transgenic lines, in that the same phenomenon was observed in both the single transgene line (DGAT1 or GPD1 lines) and in the combination transgene (DGAT1 + GPD1 line). For instance, the GPD1 line had higher levels of many amino acids in stage 2, the effect which also appears in the combined DGAT1 + GPD1 line. Similarly, the DGAT1 line had higher levels of unsaturated fatty acids in stage 1, and this pattern was also observed in the DGAT1 + GPD1 line. On the other hand, some trends appeared to be present only in one of the single gene lines and the effect was not carried over to the combined DGAT1 + GPD1 line. For instance, lower levels of fatty acids were observed in the GPD1 line at stage 2, but not in combined DGAT1 + GPD1 line; whereas, higher levels of dipeptides were observed in DGAT1 line at stage 1, but not in the combined DGAT1 + GPD1 line (see Fig. 4 and Additional file 11: Table S17, Additional file 12: Table S18).

### Impact on carbon-to-nitrogen (C/N) balance and hormone profiles in Camelina seeds

**Fig. 5:**
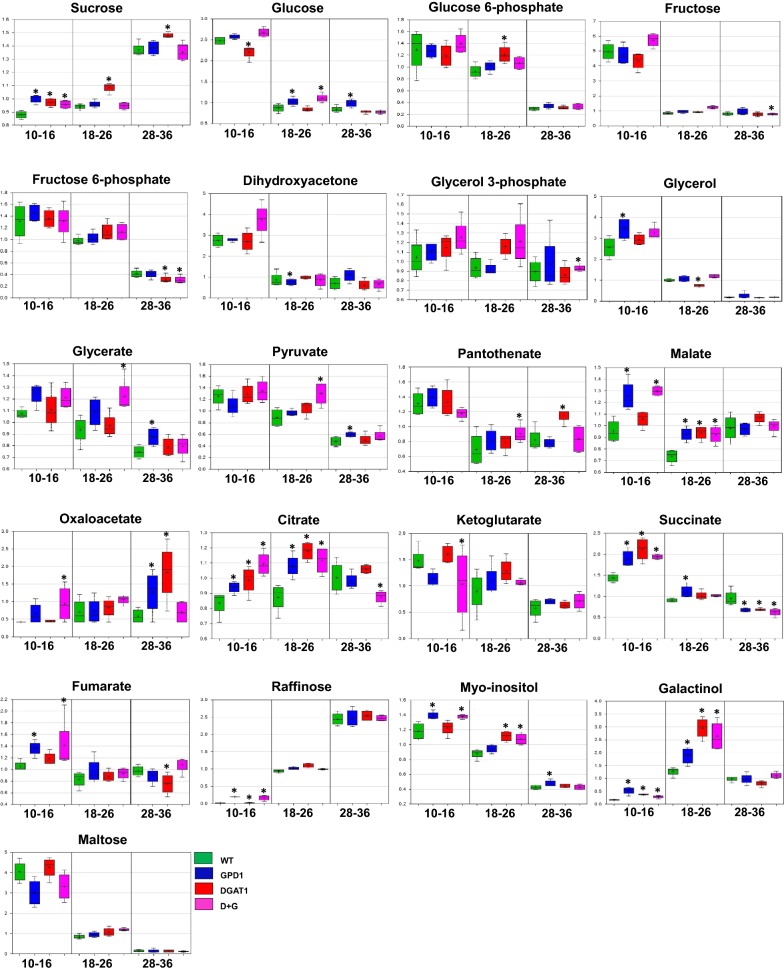
Alterations of selected metabolite levels in Camelina transgenics relative to WT seeds during seed development. Metabolites levels were determined and the relative peak areas were shown in developing seeds at 10–16, 18–26, and 28–36 DAF of wildtype (WT) and GPD1, DGAT1, and D + G lines overexpressing both *ScGPD1* and *AtDGAT1* genes. The levels of selected metabolites involved in glycolysis, TCA cycle, acetyl-CoA production, fatty acid synthesis, and TAG assembly are shown in each genotype and in three stages of seed development. The Y-axis represents the median scaled imputed data for the peak raw ion counts for each metabolite. The bars represent box plots where the mean, median, upper and lower quartiles, and the max and min distribution of values are presented. *Significant difference at *P *< 0.05 compared with the WT, based on Two-way ANOVA test

While a very large proportion of the compounds showed changes in abundance over the developmental time course, we highlighted herein a few pathways that are known to be associated with carbon flow and nitrogen metabolism, as this subject is the focus of the present study. The sucrosyl-inositol pathway (also known as the RFO, Raffinose Family Oligosaccharide pathway), which leads to the production of the storage oligosaccharides raffinose, stachyose, galactinol, etc., is important in the development of orthodox seeds as carbon stores [55]. It also serves to provide critical osmoprotectants involved in stress responses in seed and vegetative tissues [55, 56]. As expected, we observed a substantial accumulation of the sugars raffinose, stachyose, and galactinol in Camelina WT and transgenic seeds during development (Fig. 5), as these sugars are considered as primary source of carbon for the RFO pathway. We should note that the relative increase in accumulation of these sugars at earlier stages might not reflect a significant increase in the absolute levels of these metabolites because their levels were estimated to be very low in Camelina mature seeds as previously reported [57]. Also, the levels of maltose sugar, an intermediate in starch degradation, was shown to decrease over the seed stages, as did inositol, a co-reactant in the RFO pathway and the substrate for phytate (myo-inositol hexakisphosphate) production, which accumulates in seeds as a storage form of phosphorus [58]. Further, there were indications of transgenic effects on the RFO pathway. Mainly, the DGAT1-expressing lines (DGAT1 and DGAT1 + GPD1) exhibited 12–15-folds higher raffinose in seed stage 1 (metabolite ratios = 15.4 and 12.6, respectively), and the significant increases (1.4–3.2 folds) in the levels of galactinol in the GPD1, DGAT1, and DGAT1 + GPD1 lines in stages 1 and 2, relative to WT (Additional file 11: Table S17, Additional file 12: Table S18).

Abscisic acid (ABA) is associated with the induction and maintenance of seed dormancy, a process dependent on orderly and regulated cell desiccation [59]. It also plays a critical role in the regulation of seed maturation and accumulation of seed oils via induction of several enzymes involved in lipid metabolic pathways, including many transcription factors [24, 60]. The relative levels of ABA were abundant at earlier seed stages in both WT and transgenic seeds, and declined somewhat in later stages, with a noticeable increase in ABA production in the DGAT1 + GPD1 lines (metabolite ratio = 1.34 in stage 2, Additional file 11: Table S17, Additional file 12: Table S18). The critical roles of ABA in seed development and maturation as well as in seed oil accumulation, which are previously reported [24, 61, 62], could be supported by the developmental reduction patterns observed for ABA levels in both WT and transgenic seeds as observed in this study.

Furthermore, another compound differentially affected by the developmental seed stages was gibberellate (GA3), a major plant hormone required for plant growth and development and seed germination [63]. The only noticeable difference in GA3 levels was a substantial increase observed in the DGAT1 + GPD1 line in the earliest seed stage (metabolite ratio = 7.33 in stage 1, Additional file 11: Table S17, Additional file 12: Table S18). The reason for this observation is not known, but it may reflect delayed degradation of the hormone, which would be expected to be depleted during seed development and establishment of seed dormancy. The hormonal profile of the major plant hormones, ABA and GA3 presented here could link their temporal and developmental reduction to the potential roles they play in transcriptional regulation of seed maturation and oil accumulation, the observation that requires further investigation.

The transgenes, most often the GPD1 line, also tended to show increased levels of several amino acid classes relative to the WT, mainly in early seed stages (Additional file 11: Table S17, Additional file 12: Table S18). For tryptophan and lysine, this effect was apparent at all three seed stages, but for most others (tyrosine, phenylalanine, valine, glycine), it was limited to the earlier stages. The double transgene (DGAT1 + GPD1 line) typically also had elevated levels, sometimes even higher than GPD1 alone. Whether the effect resulted from increased amino acid production, or from protein turnover, is not known, but one marker of protein turnover as the post-translationally modified amino acid hydroxyproline showed a lower level. In any case, the implication is that the balance between carbon and nitrogen metabolism was affected by GPD1 expression.

We also queried the data for potential additive or synergistic interactions of the two transgenes in DGAT1 + GPD1 line. The strongest and most consistent effect involved the nitrogen-rich arginine–polyamine pathway at stage 3. The accumulation of spermidine, increasing approximately 80-fold from stage 1 to stage 3, was similar for all lines, and, thus, represents a normal seed development process. However, its precursors arginine, agmatine, and putrescine accumulated differentially in the DGAT1 + GPD1 line in stage 3 in a non-additive way (Additional file 11: Table S17, Additional file 12: Table S18). That is, these precursor compounds were either non-predicatively variable or similar to WT for the single transgene lines, but the DGAT1 + GPD1 line showed much higher levels than WT or either single gene line in stage 3. This suggests a continued production of the precursors in DGAT1 + GPD1 line, possibly a sign of nitrogen excess, whereas the WT line had down-regulated this pathway at stage 3. Spermidine did not show the effect, possibly because of a deficit of decarboxy-adenosylmethionine (decarboxylated SAM), which provides the aminopropyl group for spermidine formation. It is known that SAM decarboxylase is regulated in Arabidopsis by the energy-sensing TOR pathway [64].

### Effect of the DGAT1 and GPD1 overexpression on the flow of photosynthetic carbon into seed oils

**Fig. 6:**
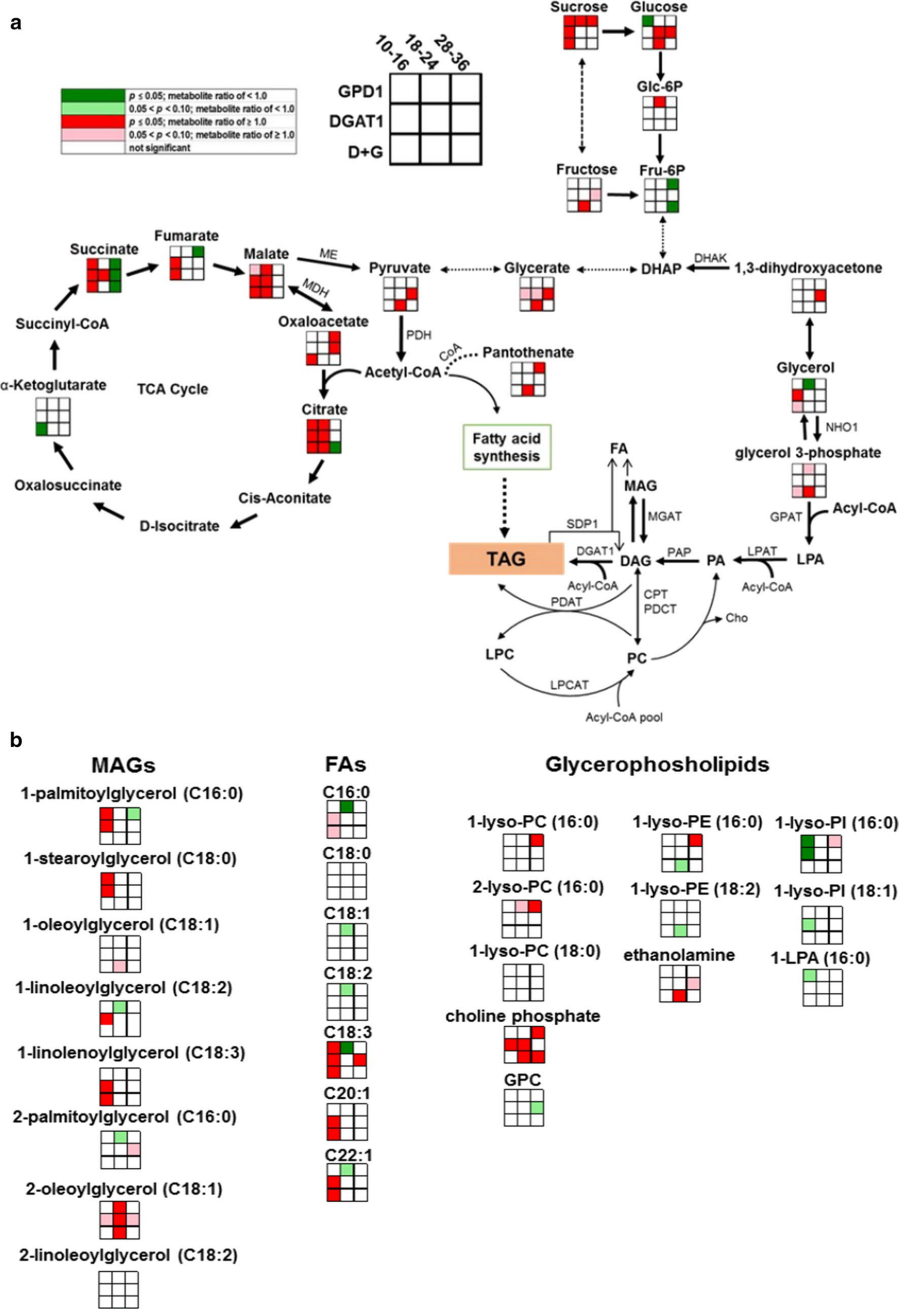
Working model for the alterations in metabolite profiling in the transgenics relative to WT seeds. The relative metabolite ratios in GPD1, DGAT1, and D + G lines as compared to WT are shown. **a** The impact of the transgenes on the metabolites involved in glycolysis, TCA cycle, fatty acid synthesis and TAG assembly and degradation, including monoacylglycerols and lysophospholipids are highlighted. **b **The impact of the transgenes on the monoacylglycerols (MAGs), fatty acids (FAs) and glycerophospholipids are highlighted. Statistical significance of the relative metabolite contents is indicated with different colors. WT, wildtype, GPD1, lines overexpressing *ScGPD1* gene, DGAT1, lines overexpressing *AtDGAT1* gene, and D + G, lines overexpressing both *ScGPD1* and *AtDGAT1* genes. The abbreviated metabolites shown are Glc-6P glucose 6-phosphate, Fru-6P fructose 6-phosphate, DHAP dihydroxyacetone phosphate, LPA lysophosphatidic acid, PA phosphatidic acid, PC phosphatidylcholine, LPC 2-lysophosphatidylcholine, DAG diacylglycerol, TAG triacylglycerol, MAG monoacylglycerol, FA fatty acids; C16:0 palmitic acid, C18:0 stearic acid, C18:1 oleic acid, C18:2 linoleic acid, C18:3 α-linolenic acid, C20:1 gondoic acid, C22:1 erucic acid, 1-lyso-PC (16:0) lyso-phosphatidylcholine with 16:0 at *sn*-1 position (1-palmitoyllysophosphatidylcholine), 1-lyso-PC (18:0) lyso-phosphatidylcholine with 18:0 at *sn*-1 position (1-stearoyl lyso-phosphocholine), 2-lyso-PC (16:0) lyso-phosphatidylcholine with 16:0 at *sn*-2 position (2-palmitoylglycerophosphocholine), GPC glycerophosphorylcholine, 1-lyso-PE (16:0) lyso-phosphatidylethanolamine with 16:0 at *sn*-1 position (1-lysophosphatidylethanolamine), 1-lyso-PE (18:2) lyso-phosphatidylethanolamine with 18:2 at *sn*-1 position (1-linoleoylglycerophosphoethanolamine), 1-lyso-PI (16:0) lyso-phosphatidylinositol with 16:0 at *sn*-1 position (1-palmitoylglycerophosphoinositol), 1-lyso-PI (18:1) lyso-phosphatidylinositol with 18:1 at *sn*-1 position (1-oleoylglycerophosphoinositol), 1-LPA (16:0) 1-palmitoylglycerophosphoglycerol. The abbreviated enzymes shown are NHO1 glycerol kinase, GPAT glycerol 3-phosphate acyltransferase, LPAT lysophospholipids acyltransferase, PAP Phosphatidate phosphatase, LPCAT lysophosphatidylcholine acyltransferase, DHAK dihydroxyacetone kinase, SDP1 triacylglycerol lipase, DGAT1 diacylglycerol acyltransferase 1, PDCT phosphatidylcholine: diacylglycerol cholinephosphotransferase, CPT CDP-choline: diacylglycerol cholinephosphotransferase, MGAT monoacylglycerol acyltransferase, PDH pyruvate dehydrogenase, MDH malate dehydrogenase, ME Malic enzyme

To illustrate the biochemical changes that control the metabolic flow of photosynthetic carbon into TAGs accumulated in Camelina seeds, we highlighted the relative metabolite content of several key metabolites of glycolysis, the TCA cycle, acetyl-CoA production, fatty acid synthesis, and TAG assembly and accumulation (Fig. 5). Accordingly, we created a working model to emphasize how these metabolites from distinct pathways led to more oil accumulation in Camelina transgenics (Fig. 6). Our results showed that overexpression of DGAT1 and/or GPD1 has significantly impacted sucrose (Suc) metabolism, the primary source of carbon, in addition to glucose and fructose, for ATP and reductants utilized by plant embryos for fatty acid synthesis. Suc cleavage would provide more sugars to stimulate lipid synthesis [65, 66]. Overall, the levels of sucrose were slightly, but significantly, increased in the GPD1 line during seed development (metabolite ratios were 1.11, 1.15, and 1.08 in seed stages 1, 2, and 3, respectively). But, in both DGAT1 and DGAT1 + GPD1 lines, Suc levels were only increased at the early seed stage (10–16 DAF, metabolite ratios were 1.14 and 1.09, respectively). Sucrose is mostly cleaved by the activity of the two enzymes, sucrose synthase (SUS, EC 2.4.1.13) and invertase (INV, EC 3.2.1.26), and the cleaved products are metabolized through glycolysis [65]. It is not clear to us from the observed sucrose levels whether the sucrose cleavage is a main route in producing precursors for increased fatty acid synthesis or the slight increase in sucrose in the transgenic seeds is instead due to a backup in carbon metabolism. Relatively, as we have observed from the transcripts profile, neither sucrose synthases nor invertases showed significant changes in transgenic seeds relative to WT (Table 1), and coincidently, a few plant invertase inhibitors were among the transcripts which shown to be up-regulated in GPD1 or DGAT1 lines (Additional file 3: Table S3, Additional file 5: Table S5, Additional file 7: Table S7, Additional file 9: Table S9).We also noticed an associated increase in Glc levels, particularly in DGAT1 line, with no significant changes in glucose 6-phosphate (G6P) or fructose levels, but a significant reduction (~ 25% decrease) in fructose 6-phosphate (F6P) levels. This could result from the subsequent exchange between F6P and dihydroxyacetone phosphate (DHAP) to stimulate fluxes into pyruvate metabolism. The plastidic acetyl-CoA is mainly synthesized from pyruvate via the pyruvate dehydrogenase activity in the plastid. The relative content of PYR in GPD1 line was similar to WT, but it was significantly increased in DGAT1 line, relative to WT (metabolite ratios were 1.24 and 1.47 in DGAT1 and DGAT1 + GPD1 lines, respectively (Figs. 5, 6, and Additional file 11: Table S17, Additional file 12: Table S18). Since there are evidences reported previously to support the finding that plastidic PYR is a precursor of acetyl-CoA [65, 67], we expected increased acetyl-CoA and, therefore, increased fatty acid synthesis rates in plastids of Camelina transgenics. This expectation should be based on whether the activity of mitochondrial pyruvate dehydrogenase in transgenic seeds is reasonable to stimulate acetyl-CoA production, the precursor for fatty acid synthesis, and ultimately stimulate lipid deposition in developing seeds [68]. However, the relationship between the acetyl-CoA pool size and the flux into fatty acid/TAG was not observed in the study by Schwender et al. [68]. In our current study, neither the expression of pyruvate dehydrogenase, and ATP citrate lyase, nor the acetyl-CoA carboxylase genes was changed in response to DGAT1 or GPD1 overexpression. Moreover, unlike the high expression levels detected for pyruvate dehydrogenase and ATP citrate lyase in Camelina seeds, the acetyl-CoA carboxylase was expressed in lower abundance, which could be a potential limitation to stimulate fatty acid production into plastids. Even though, our analysis is quite general rather than organelle specific to emphasize the contribution of plastidic or cytosolic glycolysis to provide the required pyruvate for fatty acid synthesis in developing Camelina seeds.

Further, since carbohydrates and fatty acid metabolism requires providing Coenzyme-A (CoA) particularly during storage compound accumulation, we also highlighted the metabolite content of the pantothenate (vitamin B5), an essential precursor of CoA and acyl-carrier protein synthesis [67]. The content of pantothenate was significantly decreased during seed development in both WT and transgenics lines (metabolite ratios were ranged from 0.53 to 0.77), which could indicate its developmental utilization to support the demands and homeostasis of CoA in seeds. Moreover, there was an obvious positive impact on pantothenate levels in Camelina transgenics, relative to WT. Overexpressing GPD1 in GPD1 or DGAT1 + GPD1 lines has substantially increased the relative content of pantothenate (metabolite ratios were 1.4 and 1.3, respectively, Figs. 5, 6, and Additional file 11: Table S17, Additional file 12: Table S18). Since CoA is acetylated to acetyl-CoA through glycolysis via sugar breakdown and through β-oxidation via fatty acid breakdown, or from ketogenic amino acid degradation [69], an increase in pantothenate content could indirectly increase the levels of acetyl-CoA, the precursor for fatty acid synthesis, and thus stimulating lipid synthesis in transgenic Camelina seeds.

The resulting acetyl-CoA can feed into FA synthesis pathways or be incorporated into the TCA cycle to maintain a cyclic flux mode in which the metabolite content of all the cycle intermediates remains constant. The TCA cycle takes place in the mitochondria, and it begins with the condensation of oxaloacetate (OAA) and acetyl-CoA, oxidizing organic carbon substrates to produce the reducing equivalents, NADH, and FADH_2_, that provide ATP synthesis via oxidative phosphorylation [69]. To monitor the flux into TCA, we reported the relative metabolite alterations in the levels of citrate, α-ketoglutarate, succinate, fumarate, malate, and oxaloacetate in Camelina transgenic seeds relative to that in WT. As expected, the TCA cycle-related metabolites were accumulated in higher abundances in Camelina transgenics compared to their levels in WT. The levels of citrate were significantly higher in GPD1, DGAT1, and DGAT1 + GPD1 lines (metabolite ratios were 1.35, 1.24, and 1.28, respectively) particularly in seed stage 2, relative to WT. Also, there were obvious impacts on the levels of succinate, fumarate, and malate in the transgenic seeds. The levels of succinate have increased significantly at early seed stages in the three transgenics, relative to WT (metabolite ratios were ~ 1.48, 1.33, and 1.35 in GPD1, DGAT1, and DGAT1 + GPD1, respectively), and then its levels were significantly decreased at later seed stages, probably due to the flux into fumarate and malate production. The levels of fumarate were shown to increase slightly, but significantly, in DGAT1 and DGAT1 + GPD1 lines at seed developmental stage. This increase was correlated with the observed significant increases in malate levels in seed stages 1 and 2 in these lines (metabolite ratios were 1.32 and 1.26 in DGAT1, 1.36 and 1.25 in DGAT1 + GPD1, respectively) and with the increase in oxaloacetate levels in the later seed stages (metabolite ratios were ~ 3.12, 2.15, and 2.24 in GPD1, DGAT1, and DGAT1 + GPD1, respectively, Figs. 5, 6, and Additional file 11: Table S17, Additional file 12: Table S18). The positive impacts on TCA cycle intermediates highlighted herein could suggest the existence of the conventional cyclic flux mode of TCA to provide more carbon pools and increased overall energy status (i.e., higher ATP synthesis rates) in developing seeds for lipid accumulation and biomass production in Camelina transgenics more than that in WT seeds.

Nonetheless, it was also reported that TCA cycle can be active in non-cyclic flux mode, with or without acetyl-CoA as an input, to support other functions as to provide carbon skeletons for metabolic processes and to metabolize organic acids produced in other pathways where the demands for ATP is low or if alternative sources of ATP exist [69]. For instance, the TCA metabolism can be established to support carbon skeletons for nitrogen assimilation (the flux from acetyl-CoA to α-ketoglutarate) and aspartate biosynthesis (production of OAA from malate) rather than to synthesize ATP as previously reported in the flux-balance model of the heterotrophic Arabidopsis metabolism [70]. A similar scenario probably exists in the Camelina transgenics, analyzed in the current study, where the TCA cycle acts to provide carbon pools for amino acid metabolism via α-ketoglutarate or via malate-to-OAA conversion as there was evidence of the impact on the nitrogen metabolism discussed above in transgenic Camelina seeds. Further studies should be conducted to confirm this possibility.

It was reported that the cyclic flux mode of TCA was completely missing in the canola (oilseed rape) embryos cultured on medium supplemented with glutamine and alanine as the nitrogen source [71]. There was a small and reversed flux from 2-oxoglutarate to citrate, a considerably higher forward flux from 2-oxoglutarate to malate/OAA, and a large flux from malate/OAA to citrate. Respectively, the acetyl-CoA which is required for fatty acid elongation is produced from citrate in the cytoplasm via ATP citrate lyase, and the resulting OAA re-enters the mitochondria to support OAA-to-citrate conversion. In this scenario, the role of the TCA cycle is to support fatty acid synthesis with the precursors more than generating ATP demands for biosynthesis.

Considering malate as a key intermediate in the plastidic biosynthesis of fatty acids, which can supply the required NADPH and PYR [71], its increased levels in the transgenic seeds could be the reason for the relatively higher PYR content (see Figs. 5, 6, and Additional file 11: Table S17, Additional file 12: Table S18). The increased levels for malate in the transgenic seeds could be correlated to the slight increases in transcript levels of phosphoenolpyruvate (PEP) carboxylase, but not in malate dehydrogenases, as observed in Additional file 1: Table S13. Therefore, we speculate that the higher acetyl-CoA could stimulate the cyclic flux into TCA or feed into FA synthesis and elongation pathways. This metabolic fate of malate is proposed in *B. napus* embryos where malate is produced into the cytoplasm via the activities of both cytosolic PEP carboxylase (EC 4.1.1.31) and malate dehydrogenase (EC 1.1.1.37), and then it enters the plastids to supply NADPH and PYR to the plastidic synthesis of FAs [64]. However, the contribution of malate and oxaloacetate-derived metabolites to plastidic fatty acid synthesis was quite small as compared to the alternative metabolites, i.e., glucose 6-phosphate, PYR, and dihydroxyacetone phosphate (DHAP), as indicated from previous analyses using the metabolic flux [72] and the isotope dilution experiments [73].

Camelina, similar to many other plants, can use different routes to synthesize glycerol 3-phosphate (G3P), the substrate needed to supply the backbone for TAG synthesis. G3P can be produced directly from the DHAP via GPD1, or it can be synthesized from glycerol via glycerol kinase [74]. We addressed the impact of overexpressing GPD1 and/or DGAT1 on the production of G3P in Camelina seeds, and the results indicated no difference in metabolite contents of G3P in GPD1 or DGAT1, but a slight increase observed in DGAT1 + GPD1 line in seeds at stage 2 (metabolite ratio was 1.29), relative to WT (Figs. 5, 6, and Additional file 11: Table S17, Additional file 12: Table S18). The impact on G3P due to the transgenics could be present but was not detectable, maybe because of the quick utilization or exchange between G3P and glycerol, or the potential downstream flux into lysophosphatidic acid (LPA). To support these assumptions, the data from transcripts profile (Additional file 1: Table S13) have indicated some changes in G3P phosphatase, which hydrolyzes G3P into glycerol, or changes in lysophosphatidyl acyltransferases (LPAT 4 and LPAT5) in response to DGAT1 or GPD1 overexpression. Even though, the transcripts data showed no changes in the levels of glycerol kinases or in levels of the indigenous GPD transcripts, but an associated negative impact on G3P acyltransferases (GPAT5 and GPAT6) as observed in Additional file 1: Table S13. Coincidently, the detected levels of G3P in WT or transgenic seeds were similar with no significant changes observed during the seed development (from day 10 through day 36 after flowering). This could indicate an expeditious exchange between G3P and its related metabolites or could suggest that the G3P production is somewhat limited in Camelina seeds. We also believe that understanding the regulation of G3P-related genes seems to be critical to regulate the cellular levels of G3P, a metabolic intermediate of lipid, glucose, and energy metabolism. Furthermore, the metabolite contents for the dihydroxyacetone (DHA) and glycerol, the potential precursors for G3P, were shown to be developmentally decreased in both WT and transgenic lines, which could also indicate a quick developmental and temporal utilization of these intermediates in seeds. There were no changes in the levels of DHA in the transgenic seeds relative to WT, except for a significant increase in DGAT1 line in seeds at stage 3 (metabolite ratio was 1.55). We also noticed a significant increase in the levels of glycerol in the transgenic DGAT1 line at early seed stage (metabolite ratio was 1.34), but it is not clear whether or not the change occurred in DHA and glycerol levels will be translated into a change in G3P levels (see Figs. 5, 6, and Additional file 11: Table S17, Additional file 12: Table S18). Since in the present study, we have not measured the contents of DHAP, a precursor for GPD1, we could not directly link the metabolic changes occurred upstream G3P with its content, and resolve whether or not G3P production would stimulate lipid synthesis in seeds. Besides, it should be noted that the reported metabolite contents are not organelle-specific, but overall relative values and may not represent the absolute quantity in the cytosol or plastid. Therefore, there is a need to measure the subcellular metabolite levels to understand the oilseed metabolism better.

The impact of GPD1 and/or DGAT1 overexpression on lipid-related metabolites was also addressed in the current study. The relative metabolite contents of glycerolipids and phospholipids, including free fatty acids (FFAs) were quantified in Camelina WT and transgenic lines (Fig. 6 and Additional file 11: Table S17, Additional file 12: Table S18). The results indicated that the DGAT1 overexpression, in the single as well as in combination with GDP1, was associated with the accumulation of unsaturated fatty acids and some monoacylglycerols (MAGs), particularly in seeds at early stages of development. These included fatty acids of different chain lengths (C:18 to C:24), and varying levels of unsaturation, including linolenate, eicosenoate, docosadienoate, and nervonate, among others, which reflect the general fatty acid makeup of Camelina. Further, the affected MAGs included the C:16 and C:18 species (with 1, 2, and 3 double bonds) in DGAT1 and GPD1 lines, particularly at early stages of seed development. Due to the fact that we did not detect DAGs in the analysis platform used in the current research, we have no idea whether or not they correlate with MAGs. This DGAT1-related effect on lipids was not seen in later stages. In fact, all the transgenic lines, including WT, tended to have higher FFAs and MAGs in seed stage 2, and lower levels in seed stage 3. The increased accumulation observed for the FFAs in DGAT1 line indicates the possibilities that (i) fatty acid synthesis rates increased at early seed stages via increased DGAT1 activity, (ii) these free fatty acids were not incorporated into MAG, DAG, and TAG or iii) degradation of TAG or DAG, due to lipase reactions at early seed stages generated FFAs and MAGs. Unlike the impact on FFAs and MAGs observed in Camelina transgenics, the levels of lysophospholipids, including some lysophosphatidylethanolamines, lysophosphatidylcholines, and lysophosphatidylinositols, did not change, but a slight increase in choline phosphate, an intermediate in the synthesis of phosphatidylcholine, was observed (Figs. 5, 6, and Additional file 11: Table S17, Additional file 12: Table S18).

## Conclusions

The data obtained from the transcriptomic and metabolomic profiling of Camelina WT seeds [75] have allowed us to select many candidate genes/enzymes to be manipulated via genetic engineering approaches to increase seed and oil yields in Camelina, and accordingly, we initially targeted two enzymes in TAG synthesis pathway; GPD1 and DGAT1. Combining the overexpression of the genes encoding these two enzymes in Camelina transgenic lines has led to positive effects on seed and oil yields, as compared to the WT plants [12]. However, to understand the molecular and biochemical consequences of increasing seed oil in Camelina and to enhance the seed and oil production further, we needed to identify the metabolic bottlenecks that affect the TAG synthesis and accumulation in seeds.

To this end, we carried out comprehensive transcript and metabolite profiling of Camelina GDP1 and DGAT1 seeds during development. The comparative transcriptome analysis of WT and transgenics has revealed temporal and developmental regulation of a large group of transcripts acting in various functional categories, with many of them controlling alternative metabolic routes in fatty acid synthesis, TAG assembly, and TAG degradation, and several encode transcriptional regulators of many seed processes. These findings are consistent with previous reports that increased DGAT levels may cause secondary regulatory effects [24, 76]. Nonetheless, there are no available reports to address the impact on transcript profiles in response to increased GPD levels in seeds. The metabolite profiling of Camelina WT and transgenic seeds indicated major metabolic switches, which are mainly associated with significant changes in the glycolytic and TCA intermediates, glycerolipids, including FAs, MAGs, and most amino acids, suggesting potential effects on carbon/nitrogen balance in transgenic Camelina seeds.

In the current research, we tried to compare the RNA-Seq and metabolome datasets and infer the relative decreased or increased metabolic changes from transcript profiles in Camelina transgenic seeds, but it seems a speculative attempt due to the multiple regulatory steps involved, including gene expression regulation, protein synthesis and turnover, enzymatic activities, and reaction fluxes. Further, we also need to consider the notion that transcript abundance on its own could not infer activity/flux in the major metabolic pathways [77]. However, this study has led to the identification of novel target transcripts worthy to be further investigated through genetic engineering and gene stacking approaches to generate Camelina transgenics with improved seed and oil qualities. The transcript profiles of Camelina seeds indicated significant changes in the regulation of a large group of transcription factors, and the metabolite profiles exhibited associated major changes in glycolysis and TCA intermediates as well as fatty acid synthesis precursors and TAG, specifically hydrolysis, in response to DGAT1 and/or GPD1 overexpression. Notably, as we observed from the transcript profiles (see Additional file 1: Table S13), the expression of DGAT1 and GPD1 was associated with increases in transcript levels of genes encoding lipid transfer proteins, involved in TAG assembly (i.e. GPATs, LPATs, and PAPs), fatty acid synthesis precursors (i.e. pyruvate metabolism), and TAG lipases and phospholipases. However, negative impacts were also observed, in response to DGAT1 and GPD1 expression, which are associated with decrease in the transcript levels of genes involved in fatty acid synthesis (mainly 3-ketoacyl-CoA synthases), fatty acid desaturases (i.e. FAD2 and FAD3), and the oil bodies’ proteins, oleosins (particularly, oleosin 4 and oleosin 5). Based on these findings, we can conclude that TAG accumulation could be limited by: (1) utilization of fixed carbon from the source tissues as supported by the increase in glycolysis intermediates and decreased transcripts levels of transcription factors controlling the flow of carbon into seed lipids and (2) the activity of lipases/hydrolases that hydrolyze TAG pools and TAG precursors, which is supported by the increase in free fatty acids and MAGs, and the associated decrease in the oil bodies-forming proteins, oleosins. The synthesis of acetyl CoA, and acyl-carrier protein could be another limitation in Camelina transgenics. Accordingly, our research strategy to further increase seed and oil yields in Camelina will depend mainly on utilizing genetic and metabolic engineering to increase the metabolic flux through glycolytic intermediates toward increasing fatty acid synthesis in plastids. This can be achieved by targeting candidate transcription factor such as the AP2/ERWEBP ethylene-responsive transcription factor (namely, Wrinkled 1 WRL1), which controls carbon flow from sucrose import to oil accumulation in developing seeds. Further, the relative increases in MAGs and FFAs levels in the transgenics at early seed stages, as indicated from the metabolite profiles, in association with the expression of many candidate transcripts involved in fatty acid synthesis and breakdown, highlight the need to create metabolic sinks. This could be achieved by increasing the flux into DAG accumulation, utilizing MAG and/or phospholipids, i.e., phosphatidylcholine as precursors by targeting genes such as the lysophospholipase 2, a MAG acyltransferase (MGAT) homologous and the Phosphatidic acid phosphatase-related/PAP2-related protein, which is a PDCT homologous. Further, we believe that the oil packaging in Camelina transgenic seeds seems to be affected by the downregulation of the oleosins (Ole 4 and Ole 5, see Additional file 1: Table S13), in response to DGAT1 or GPD1 expression. We will consider utilizing oleosins in the future research to improve Camelina seed abilities to fit the excess oil accumulation and provide precursors for TAG accumulation, considering the previous finding that some oleosins (i.e. Ole 4) can also act as a MAG acyltransferase or a phospholipase A2, thus utilizing MAG or phospholipids to build DAG and TAG [76]. Moreover, to prevent TAG hydrolysis, two candidate TAG lipases can be targeted (namely, SDP1 and TLL1) through knock-down studies. A list of candidate genes identified as limitations is provided in Table 2. Finally, since increasing oil and seed production in Camelina and other crops is always limited by carbon flux from the source tissues, and considering this as a challenge we faced in conducting this study, metabolic flux analysis (MFA) and metabolic control analysis (MCA) [78], in a combination with transcriptomic analysis will be considered in future research to better understand the carbon allocation and to target the flux toward seed biomass and oil synthesis pathways. Another challenge that needs to be addressed is to more efficiently link/integrate the transcriptome and metabolome data, rather than just link the information derived from these analyses, and this can be achieved once there is an enriched database of omics data for Camelina with improved annotation. Collectively, this study led to the identification of novel target transcripts worthy to be further investigated through genetic engineering and gene stacking approaches to generate Camelina transgenics with improved seed and oil qualities.

**Table 2:** A list of candidate genes identified as limitations for further improve Camelina for better seed and oil qualities

Gene name	Gene ID	Gene description
WRl1	Csa06g028810 Csa04g040400 Csa09g064030	WRINKLED1, encodes transcription factor of the AP2/ERWEBP
MGAT	Csa17g092850 Csa03g059790 Csa17g092830	Lysophospholipase 2, encodes a monoacylglycerol acyltransferase
Ole 4	Csa04g015780 Csa09g014800 Csa06g008780	Oleosin 4
Ole 5	Csa19g001360 Csa005325200 Csa10g042420	Oleosin 5
PDCT	Csa19g022610 Csa15g020460 Csa01g018440	Phosphatidic acid phosphatase-related/PAP2-related, encodes a phosphatidylcholine:diacylglycerol cholinephosphotransferase
ACC1	Csa14g047290 Csa03g039320 Csa05g058160	Acetyl-CoA carboxylase
PLA2-Alpha	Csa15g059780 Csa01g038410 Csa19g040900	Phospholipase A2-alpha
SDP1	Csa13g006100 Csa08g060480 Csa20g005210	Triacylglycerol lipase, sugar-dependent1
TLL1	Csa14g049770 Csa17g071380 Csa03g047400	Triacylglycerol lipase-like 1

## Methods

### Plant material

Camelina sativa (cultivar: Suneson) was grown on soil under controlled environmental conditions in greenhouse. The conditions were 21 °C/day and 18 °C/night in a 16-h-day/8-h-night photoperiod at a light intensity of 400 µmol photons m^−2^ s^−1^, and humidity ranges from 30 to 40%. Plants were watered regularly and were fertilized with 200 ppm N of Peters Professional 20-10-20 Peat-lite water-soluble fertilizer once a week. During inflorescence, the emerging flowers were marked, and at given time intervals following flowering (10–15 and 16–21 DAF for RNA-Seq; 10–16, 18–26, and 28–36 DAF for metabolite profiling) seeds were collected and immediately frozen in liquid N, and stored at − 80 °C. These sampling periods were selected based on the oil and other storage compounds synthesis and accumulation rates as reported in previous studies [3, 25, 75, 79, 82].

Camelina plants used in the present study were representing T3 generation of homozygous transgenics, line DGAT1 #2, namely DGAT1, which is overexpressing a cDNA of Diacylglycerol acyltransferase from *Arabidopsis thaliana* (*AtDGAT1,* TAIR ID: AT2G19450.1), line GPD1 #2, namely GPD1, which is overexpressing a cDNA of NAD+-dependent glycerol-3-phosphate dehydrogenase from *Saccharomyces cerevisiae* (yeast, *ScGPD1,* NCBI Gene ID: 851539), and line DGAT1 + GPD1 #11, namely D + G, which is co-expressing *AtDGAT1* and *ScGPD1*, in addition to the nontransgenic wild-type (WT) control.

### RNA extraction, cDNA library construction, and RNA sequencing

Total RNA was extracted from Camelina seeds using the plant RNeasy mini kit (Sigma-Aldrich), according to the manufacturer’s recommendations. Purity and quantity of RNA were evaluated on Nanodrop 2000 spectrophotometer and Agilent 2100 Bioanalyzer. A total of 5 µg RNA was shipped in dry ice to the RTSF Genomics Core at the Michigan State University for cDNA libraries preparation and RNA sequencing. RNA samples were prepared for sequencing using the Illumina TruSeq Stranded mRNA Library Preparation Kit LT. Subsequently, adaptor ligation was performed, and the quality of cDNA was assessed. The libraries were then combined and loaded on HiSeq 2500 Rapid Run flow cell. Sequencing was performed on Illumina HiSeq 2500 using standard Rapid SBS reagents and procedures.

### Bioinformatics and data analysis

Base calling was performed with Illumina Real-Time Analysis (RTA) software (v1.17.21.3), and the obtained sequencing reads were demultiplexed, converted into FASTQ files by the Illumina Bcl2Fastq software (v1.8.4), and the FASTQ files were created. The reads obtained from Illumina sequencing were trimmed to remove adaptor sequences, low-quality sequence (score > 0.05), ambiguous nucleotides Ns, terminal nucleotides in both 3′ and 5′ ends, and the relatively short reads (< 40 bp). The obtained clean reads were analyzed and mapped to Camelina reference genome available from the Prairie Gold project (http://www.camelinadb.ca, Cs_genome_sequence_build_V2.0) by using CLC Genomics Workbench 7.5 (http://www.clcbio.com) according to the analysis pipeline described by [75].

RNA sequencing reads were mapped to the genes and transcripts assigned to the reference genome following the method described by [75, 80, 81]. Accordingly, the raw read counts for each Camelina transcript was normalized to gene length, library size, and number of mapped reads, which resulted in the expression value known as reads per kilobase of exon model per million mapped reads (RPKM). The original RPKM values were quantile normalized, and then log_2_ transformed. Using the obtained RPKM-normalized-log_2_-transformed values, the Principal Component Analysis (PCA), invoked on transcript level, was conducted to compare the RNA-seq data obtained from WT and transgenic lines at two stages of seed development using *covariance matrix* in CLC Genomics Workbench.

Comparative analysis of transcriptome data was conducted to determine the fold differences in gene expression levels between Camelina wild-type and transgenic lines. Statistical analysis based on Gaussian tests (CLC Genomics Workbench, http://www.clcbio.com) and EdgeR (MultiExperiment Viewer, MeV, http://www.tm4.org) pipelines was performed, and the two-sided P value and false discovery rate (FDR) values were used to estimate the significance of the differences. Genes and transcripts were defined as differentially expressed (DE) if (i) the fold change (FC) of the expression between conditions is significant (FC ≥ 1.5 or ≤ − 1.5), (ii) *P* value and/or FDR is ≤ 0.05, (iii) RPKM ≥ 0.1 (in log2 scale). The annotation of the DE genes was performed using Blast2Go server tools (http://www.blast2go.com, [82] and the GO for the transcripts was assigned using Kyoto Encyclopedia of Genes and Genomes KEGG maps (http://www.genome.jp/kegg/).

### Quantitative real-time PCR (qRT-PCR)

All qRT-PCR reactions were performed in Eppendorf Mastercycler^®^ep realplex thermal cycler using the intercalation dye ABsolute Blue QPCR SYBR Green master mix kit (Thermo Scientific) as a fluorescent reporter. All PCR reactions were performed in triplicates for three biological replicates in 25 μl volumes using 1 μl of each forward and reverse primers (25 pmol each), 12.5 μl of SYBR green master mix, 1 μl of cDNA (100 ng/μl), and 9.5 μl HPLC molecular biology grade water. RNAs and cDNAs were prepared from Camelina seeds harvested between 10 and 16 days after flowering (DAF), and PCR products were quantified, using specific PCR primers for the gene of interest, in the qPCR cycling program of 1 cycle at 95 °C for 15 min, 30–40 cycles at 95 °C for 15 s, 50–60 °C for 30 s, and 72 °C for 30 s. The quantification of PCR products was performed using the 2^−ΔΔ*Ct*^ method [83], and the Camelina β-actin gene was used as internal reference to normalize the relative amount of mRNAs for all samples. The error bars represent the standard errors for the fold changes of relative gene expression calculated from at least two independent biological replicates and triplicate PCR reactions for each sample. A list of PCR primers used is presented in Additional file 1: Table S16.

### Metabolite analyses

Metabolome analysis was performed at the Metabolon, Inc (http://www.metabolon.com) under the project number BOAH-0102-13VW, and the samples were extracted and prepared for analysis using Metabolon’s standard solvent extraction method. In brief, samples were prepared using an automated MicroLab STAR^®^ system (Hamilton Company, UT, USA). The samples were extracted using a solvent of 80% methanol. To remove proteins and their bound molecules, and to recover chemically diverse metabolites, proteins were precipitated with methanol by shaking for 2 min in the presence of glass beads using a Geno/Grinder 2000 (Glen Mills, Inc. NJ, USA). After each extraction, the sample was centrifuged and the supernatant removed using the MicroLab STAR^®^ automated system, followed by re-extraction of the pellet. The resulting extracts were pooled and then split into four equal aliquots, one for UPLC–MS/MS with positive ion mode electrospray ionization, one for analysis by UPLC–MS/MS with negative ion mode electrospray ionization, one for GC–MS, and one sample was reserved for backup. Aliquots were placed briefly on a TurboVap^®^ (Zymark, Runcorn, UK) to remove the organic solvent, frozen, dried under vacuum, and then prepared for the appropriate instrument.

### LC–MS/MS and GC/MS analysis

For LC–MS/MS analysis, extract aliquots were reconstituted in acidic conditions and were gradient eluted using water and methanol containing 0.1% formic acid. The basic extracts were also gradient eluted using water and methanol containing 6.5 mM ammonium bicarbonate. LC–MS/MS was carried out using a Waters ACQUITY ultra-performance liquid chromatography (UPLC) (ThermoElectorn Corporation, CA, USA) with an electrospray ionization (ESI) source coupled to a linear ion-trap (LIT) mass analyzer. The scan range was from 80 to 1000 m/z.

For GC/MS analysis, aliquots were dried under vacuum for a minimum of 18 h, and then derivatized under dried nitrogen using bistrimethyl-silyltrifluoroacetamide (BSTFA). The derivatized samples were analyzed on a Thermo-Finnigan Trace DSQ fast-scanning single-quadrupole MS (ThermoElectorn Corporation, CA, USA) using electron impact ionization (EI) and operated at unit mass resolving power. The scan range was from 50 to 750 m/z. The aliquots were separated on a 5% diphenyl/95% dimethyl polysiloxane-fused silica column (20 m × 0.18 mm ID, 0.18 μm film thickness), and the initial oven temperature was 64° ramped to 340 °C in a 17.5-min period, and helium was the carrier gas.

### Data extraction and compound identification

Compounds were identified by automated comparison to Metabolon’s library entries of purified standards or recurrent unknown entities using appropriate proprietary software. Peaks that eluted from LC–MS/MS and GC/MS method were compared with a library based on authenticated standards that contain the retention time/index (RI), mass–charge ratio (m/z), and chromatographic data (including MS/MS spectral data) on all molecules present in the library. Further, biochemical identification of compounds was performed based on retention index within a narrow RI window of the proposed identification, accurate mass matching to the library, and the MS/MS forward and reverse scores between the experimental data and authentic standards. Furthermore, quality control (QC) and curation processes were designed to ensure accurate and consistent identification of the compounds and to remove those with system artifacts and background noise, if any, using Metabolon’s proprietary visualization and interpretation software (http://www.metabolon.com).

### Metabolite quantification, data normalization, and statistical analysis

Peaks were quantified using area-under-the-curve based on the analysis pipeline designed by Metabolon, Inc (http://www.metabolon.com). Accordingly, raw area counts for each compound in each sample were normalized to correct variation resulting from instrument inter-day tuning differences, and to remove any instrument sensitivity differences. Raw area counts for each compound were scaled to the median detected value for that compound, setting the medians equal for each day’s run. Missing values for a given compound were imputed with the minimum detected value for that compound. The resulted scaled imputed values were then log transformed to be statistically analyzed.

All statistical comparisons throughout the study were performed across the three stages of seed development for each genotype, relative to stage 1, and across genotypes at each developmental stage, relative to WT. Statistical analysis of the data was performed using ArrayStudio (http://www.omicsoft.com/array-studio/) and JMP (SAS, Inc. Statistical discovery software. http://www.Jmp.com). ANOVA contrasts were used to identify biochemicals that differ significantly between experimental groups. The effect of either genotype or developmental stage, and/or their interaction was determined by two-way ANOVA test. The false discovery rate (*q* value) and *P* value were calculated as an indication of the results’ confidence and statistical significance, respectively.

## Additional files

**Additional file 1: Fig S1.** Volcano plots of the relationship between the P value of statistical test (y-axis) and the log2 fold change (x-axis) showing the differentially expressed genes between WT and DGAT1 transgenic lines. **Fig. S2.** Volcano plots of the relationship between the *P* value of statistical test (y-axis) and the log2 fold change (x-axis) showing the differentially expressed genes between WT and GPD1 transgenic lines. **Table S1.** RNA-Seq datasets of Camelina developing seeds obtained from transgenic lines and non-transgenic wildtype. **Table S11.** List of selected DEGs showing ≥ 1.5-fold changes in expression between Camelina DGAT1 transgenics and WT plants. **Table S12.** List of selected DEGs showing ≥ 1.5-fold changes in expression between Camelina GPD1 transgenics and WT plants. **Table S13.** List of selected lipid-related genes differentially expressed in seeds of Camelina transgenic lines relative to WT. **Table S14.** List of selected genes encode transcription factors, which are differentially expressed in seeds of Camelina transgenic lines relative to WT. **Table S15.** Comparative quantification of transcript levels measured by qRT-PCR and RNA-Seq. **Table S16.** List of selected genes used in qRT-PCR analysis. Gene IDs, gene names, gene symbols, primer sequences, and size of amplification products.

**Additional file 2: Table S2.** List of differentially expressed genes in Camelina DGAT1 and GPD1 transgenic lines as compared to WT during seed development.

**Additional file 3: Table S3.** GO classification and KEGG metabolic pathways for the DEGs up-regulated in DGAT1 lines at 10–15 DAF.

**Additional file 4: Table S4.** GO classification and KEGG metabolic pathways for the DEGs down-regulated in DGAT1 lines at 10–15 DAF.

**Additional file 5: Table S5.** GO classification and KEGG metabolic pathways for the DEGs up-regulated in DGAT1 lines at 16–21 DAF.

**Additional file 6: Table S6.** GO classification and KEGG metabolic pathways for the DEGs down-regulated in DGAT1 lines at 16–21 DAF.

**Additional file 7: Table S7.** GO classification and KEGG metabolic pathways for the DEGs up-regulated in GPD1 lines at 10–15 DAF.

**Additional file 8: Table S8.** GO classification and KEGG metabolic pathways for the DEGs down-regulated in GPD1 lines at 10–15 DAF.

**Additional file 9: Table S9.** GO classification and KEGG metabolic pathways for the DEGs up-regulated in GPD1 lines at 16–21 DAF.

**Additional file 10: Table S10.** GO classification and KEGG metabolic pathways for the DEGs down-regulated in GPD1 lines at 16–21 DAF.

**Additional file 11: Table S17.** Metabolite contents in Camelina genotypes during seed development.

**Additional file 12: Table S18.** Relative metabolite content during seed development in Camelina genotype relative to WT as a control.

## Abbreviations

TAG: triacylglycerol
MAG: monoacylglycerol
DAG: diacylglycerol
DAF: days after flowering
RPKM: reads per kilobase of transcript per million mapped reads
DGAT: diacylglycerol acyltransferase
GPD: glycerol-3-phosphate dehydrogenase
TCA: tricarboxylic cycle
LCFA: long-chain fatty acids
qRT-PCR: quantitative real-time polymerase chain reaction
RNA-Seq: RNA sequencing
G3P: glycerol-3-phosphate
LPA: lysophosphatidic acid
PA: phosphatidic acid
PC: phosphatidylcholine
GPAT: glycerol-3-phosphate acyltransferase
DEGs: differentially expressed genes
FDR: false discovery rate
BLAST: basic local alignment search tool
LPAT: lysophosphatidyl acyltransferase
MGAT: monoacylglycerol acyltransferase
PDCT: phosphatidylcholine: diacylglycerol cholinephosphotransferase
WRl1: Wrinkled 1
KEGG: Kyoto Encyclopedia of Genes and Genomes
ER: endoplasmic reticulum
FFA: free fatty acids
LPC: lysophosphatidylcholine
SDP1: sugar-dependent protein
SDP1-L: sugar-dependent 1-like protein
Ole: oleosin
GO: gene ontology
DEGs: differentially expressed genes
PCA: principal components analysis
