# Supplementary material for: Comparative transcriptome and metabolome analysis suggests bottlenecks that limit seed and oil yields in transgenic Camelina sativa expressing diacylglycerol acyltransferase 1 and glycerol-3-phosphate dehydrogenase
Source: Biotechnol Biofuels. 2018 Dec 19;11:335. doi: 10.1186/s13068-018-1326-2 (PMC6299664; doi:10.1186/s13068-018-1326-2)
Supplement: Supplementary file 1 — Additional file 1: Fig S1. Volcano plots of the relationship between the P value of statistical test (y-axis) and the log2 fold change (x-axis) showing the differentially expressed genes between WT and DGAT1 transgenic lines. Fig. S2. Volcano plots of the relationship between the P value of statistical test (y-axis) and the log2 fold change (x-axis) showing the differentially expressed genes between WT and GPD1 transgenic lines. Table S1. RNA-Seq datasets of Camelina developing seeds obtained from transgenic lines and non-transgenic wildtype. Table S11. List of selected DEGs showing ≥ 1.5-fold changes in expression between Camelina DGAT1 transgenics and WT plants. Table S12. List of selected DEGs showing ≥ 1.5-fold changes in expression between Camelina GPD1 transgenics and WT plants. Table S13. List of selected lipid-related genes differentially expressed in seeds of Camelina transgenic lines relative to WT. Table S14. List of selected genes encode transcription factors, which are differentially expressed in seeds of Camelina transgenic lines relative to WT. Table S15. Comparative quantification of transcript levels measured by qRT-PCR and RNA-Seq. Table S16. List of selected genes used in qRT-PCR analysis. Gene IDs, gene names, gene symbols, primer sequences, and size of amplification products. [file 13068_2018_1326_MOESM1_ESM.docx]

**Comparative Transcriptome and Metabolome Analysis Suggest Bottlenecks that Limit Seed and Oil Yields in Transgenic *Camelina sativa* Expressing Diacylglycerol Acyltransferase 1 and Glycerol-3-Phosphate Dehydrogenase**

# Hesham M. Abdullah^1,2, ¥^, Sudesh Chhikara^1 €^, Parisa Akbari^1^, Danny J. Schnell^3^, Ashwani Pareek^4^, and Om Parkash Dhankher^1*^

^1^Stockbridge School of Agriculture, University of Massachusetts Amherst, MA 01003, USA

^2^Biotechnology Department, Faculty of Agriculture, Al-Azhar University, Cairo 11651, Egypt

^3^Department of Plant Biology, Michigan State University, East Lansing, MI 48824, USA

^4^Stress physiology and Molecular Biology Laboratory, School of Life Sciences, Jawaharlal Nehru University, New Delhi 100067, India

^¥^ Current address: Department of Plant Biology, Michigan State University, East Lansing, MI 48824, USA

**^€^** Current address: Centre for Biotechnology, Maharshi Dayanand University, Rohtak 124001, India

^*^Corresponding Author:

Om Parkash Dhankher
Email: parkash@umass.edu
Tel: 413-545-0062
Fax: 413-545-1058


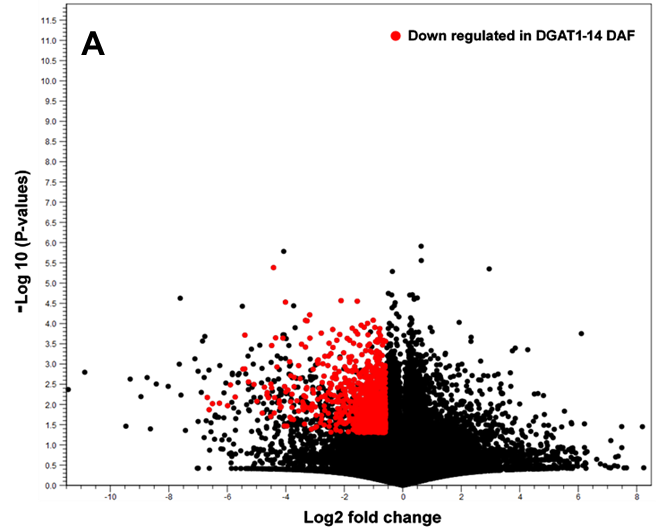

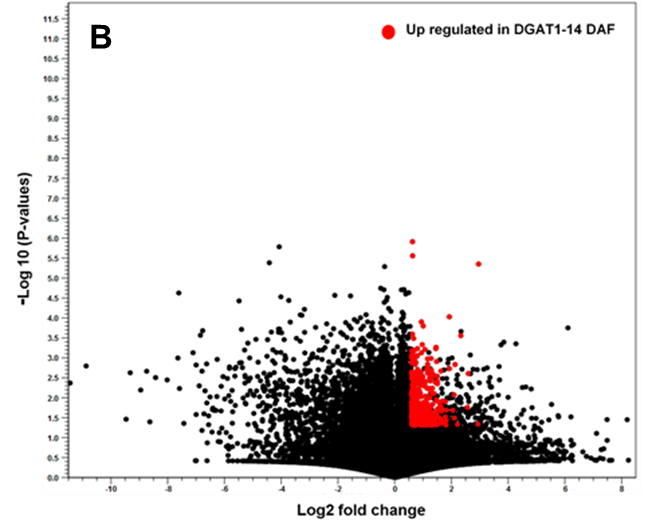

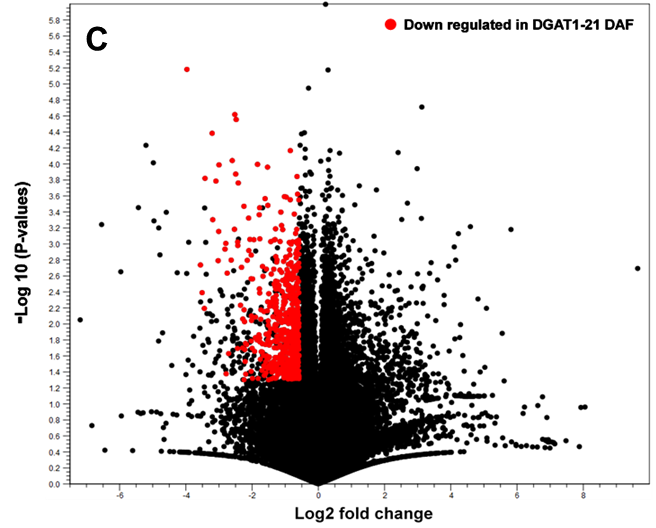

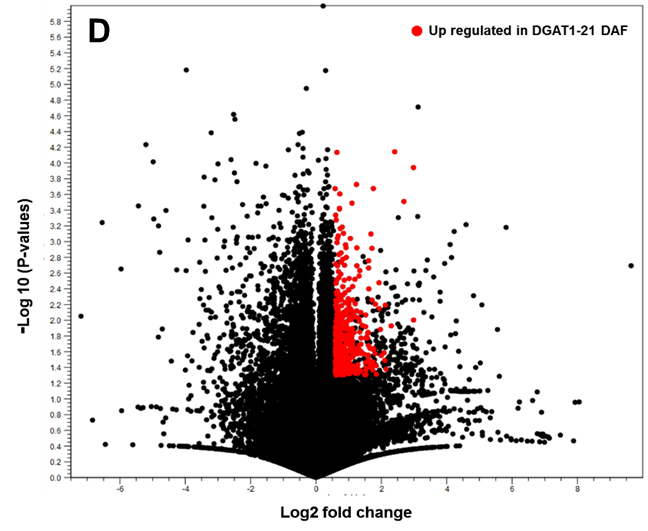


**Fig. S1.** Volcano plots of the relationship between the P-value of statistical test (y-axis) and the log2 fold change (x-axis) showing the differentially expressed genes between WT and DGAT1 transgenic lines. The genes that showed significant difference (P-value ≤ 0.05) and fold change (log2 fold change ≥ 1.5 or ≤ -1.5) were presented as red spots. A total of 1,057 and 599 genes down regulated in DGAT1 lines at 10-15 DAF (A) and at 16-21 (C), respectively, whereas a total of 448 and 415 genes were up regulated at 10-15 DAF (B) and at 16-21 DAF (D), respectively.


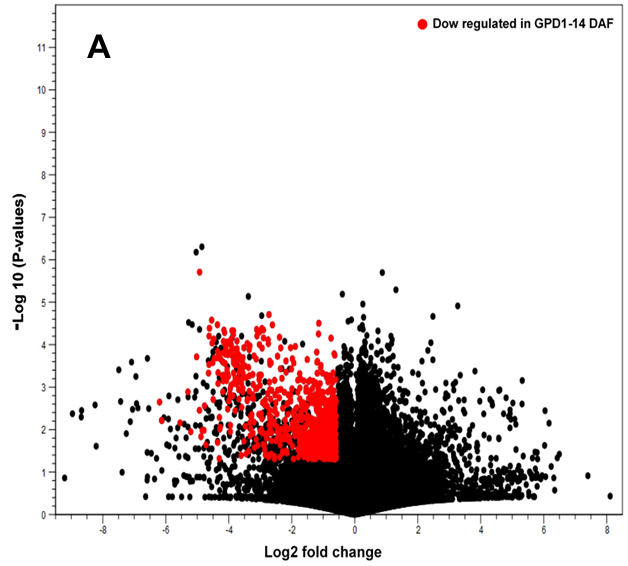

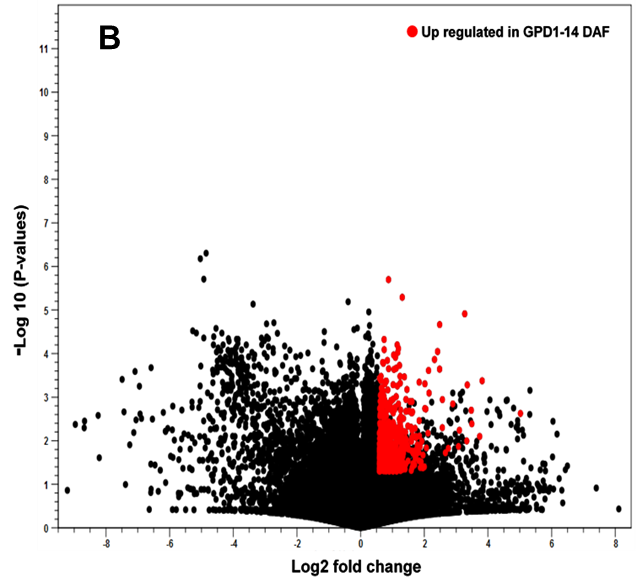

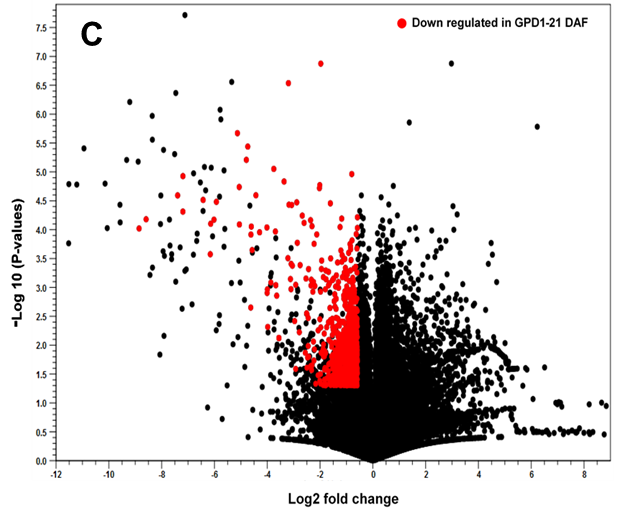

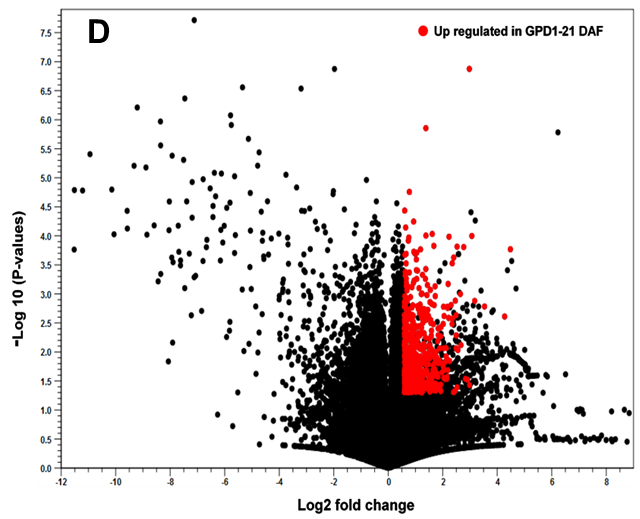


**Fig. S2.** Volcano plots of the relationship between the P-value of statistical test (y-axis) and the log2 fold change (x-axis) showing the differentially expressed genes between WT and GPD1 transgenic lines. The genes that showed significant difference (P-value ≤ 0.05) and fold change (log2 fold change ≥ 1.5 or ≤ -1.5) were presented as red spots. A total of 981 and 828 genes down regulated in DGAT1 lines at 10-15 DAF (A) and at 16-21 (C), respectively, whereas a total of 617 and 710 genes were up regulated at 10-15 DAF (B) and at 16-21 DAF (D), respectively.

| **Table S1**. RNA-Seq datasets of Camelina developing seeds obtained from transgenic lines and non-transgenic wildtype. | | | | | | |
| --- | --- | --- | --- | --- | --- | --- |
|  | WT (10-15 DAF) | | | WT (16-21 DAF) | | |
|  | B1 | B2 | B3 | B1 | B2 | B3 |
| Number of sequence reads | 53.0 M | 54.8 M | 78.9 M | 62.0 M | 61.7 M | 46.3 M |
| Number of reads after trimming | 52.0 M | 53.7 M | 76.2 M | 60.7 M | 60.5 M | 45.4 M |
| Reads mapped in pairs | 44.8 M | 45.6 M | 67.3 M | 50.7 M | 51.1 M | 38.6 M |
| Reads mapped in broken pairs | 5.9 M | 6.7 M | 8.4 M | 8.4 M | 7.9 M | 5.8 M |
| Reads not mapped | 210,281 | 241,410 | 372,878 | 319,246 | 286,780 | 184,244 |
|  |  |  |  |  |  |  |
|  | DGAT1 #2 (10-15 DAF) | | | DGAT1 #2 (16-21 DAF) | | |
|  | B1 | B2 | B3 | B1 | B2 | B3 |
| Number of sequence reads | 65.6 M | 46.2 M | 92.0 M | 36.5 M | 44.3 M | 49.1 M |
| Number of reads after trimming | 64.3 M | 44.7 M | 89.2 M | 35.4 M | 42.9 M | 47.5 M |
| Reads mapped in pairs | 58.2 M | 38.0 M | 75.5 M | 29.4 M | 35.7 M | 39.3 M |
| Reads mapped in broken pairs | 4.7 M | 5.0 M | 10.5 M | 4.6 M | 5.5 M | 6.2 M |
| Reads not mapped | 302,337 | 211,920 | 397,937 | 322,801 | 363,930 | 437,744 |
|  |  |  |  |  |  |  |
|  | GDP1 #2 (10-15 DAF) | | | GDP1 #2 (16-21 DAF) | | |
|  | B1 | B2 | B3 | B1 | B2 | B3 |
| Number of sequence reads | 97.9 M | 47.1 M | 64.4 M | 34.5 M | 41.7 M | 97.9 M |
| Number of reads after trimming | 95.5 M | 45.6 M | 63.1 M | 33.8 M | 40.5 M | 95.5 M |
| Reads mapped in pairs | 73.1 M | 38.9 M | 54.4 M | 28.8 M | 33.9 M | 73.1 M |
| Reads mapped in broken pairs | 9.0 M | 5.0 M | 7.2 M | 3.9 M | 5.1 M | 9.0 M |
| Reads not mapped | 340,610 | 190,961 | 309,764 | 281,806 | 292,694 | 340,610 |
| Shown are the two stages of camelina seed development RNA-Seq data obtained from Camelina wildtype (WT), Arabidopsis DGAT1-overexpressing line (DGAT1 #2, and Yeast GPD1-overexpressing line (GDP1 #2). Days after flowering (DAF), the number of processed reads obtained from three biological replicates (B1-B3) at different levels of data analysis of RNA sequencing (in millions M). | | | | | | |

| Table S11. List of selected DEGs showing ≥ 1.5-fold changes in expression between Camelina DGAT1 transgenics and WT plants. | | | | | | |
| --- | --- | --- | --- | --- | --- | --- |
| Gene ID | **Gene description** | **Fold change** | **P-value** | **RPKMs** | | **Regulation** |
| DEGs in DGAT1 *vs* WT | | | | | |  |
|  |  |  |  | **WT** | **DGAT1** |  |
| Csa02g065010 | non-specific lipid-transfer 4-like (LTP) | 1.51 | 2.52E-03 | 733.59 | 1105.73 | **UP** |
| Csa18g033140 | gibberellin-regulated family | 1.57 | 2.66E-02 | 609.87 | 954.80 | **UP** |
| Csa15g038220 | defensin 46 | 1.52 | 1.85E-02 | 62.24 | 94.63 | **UP** |
| Csa09g079870 | isoflavone reductase homolog P3-like | 2.43 | 1.04E-03 | 34.58 | 83.98 | **UP** |
| Csa08g055270 | merozoite surface CMZ-8-like isoform X5 | 1.60 | 4.67E-03 | 35.60 | 57.09 | **UP** |
| Csa09g009250 | low-molecular-weight cysteine-rich 45 | 1.54 | 4.52E-02 | 28.75 | 44.14 | **UP** |
| Csa05g007300 | hypothetical protein CARUB_v10024430mg | 1.62 | 1.09E-03 | 21.14 | 34.33 | **UP** |
| Csa19g034220 | PREDICTED: uncharacterized protein LOC104766284 | 1.54 | 2.55E-04 | 19.84 | 30.62 | **UP** |
| Csa08g049270 | Wound-responsive family | 1.55 | 5.89E-03 | 9.07 | 14.03 | **UP** |
| Csa08g055280 | non-specific lipid-transfer At5g64080 | 1.57 | 4.85E-02 | 7.32 | 11.50 | **UP** |
| Csa16g051630 | proline-rich extensin EPR1 | 1.77 | 1.27E-02 | 118.80 | 210.34 | **UP** |
| Csa07g063460 | UPF0540 At1g62060-like | 1.75 | 6.53E-04 | 29.84 | 52.07 | **UP** |
| Csa15g026620 | transmembrane 45A | 1.52 | 4.61E-04 | 26.44 | 40.15 | **UP** |
| Csa20g071700 | CASP 1B1 | 1.67 | 6.83E-04 | 22.01 | 36.86 | **UP** |
| Csa14g026530 | transmembrane protein | 1.64 | 4.88E-02 | 20.89 | 34.33 | **UP** |
| Csa09g075840 | kunitz-type serine protease inhibitor -like | 1.76 | 8.04E-03 | 13.03 | 22.89 | **UP** |
| Csa17g013010 | transcription repressor MYB6-like | 1.54 | 5.32E-04 | 9.89 | 15.18 | **UP** |
| Csa11g093140 | non-specific lipid-transfer 4-like (LTP) | 1.80 | 4.99E-02 | 6.34 | 11.42 | **UP** |
| Csa05g083420 | MORC family CW-type zinc finger 3-like isoform X1 | 1.58 | 4.26E-03 | 4.98 | 7.85 | **UP** |
| Csa02827s010 | actin family partial | 1.56 | 1.15E-02 | 4.89 | 7.63 | **UP** |
| Csa03g027020 | MLP 28 | 1.65 | 3.77E-04 | 4.51 | 7.43 | **UP** |
| Csa00669s010 | mannan endo-1,4-beta-mannosidase 6 isoform X3 | 1.71 | 3.79E-02 | 4.12 | 7.04 | **UP** |
| Csa11g070590 | 12S seed storage | -3.21 | 3.08E-04 | 6042.93 | 1882.38 | **Down** |
| Csa12g024730 | 2S seed storage | -2.28 | 3.53E-04 | 753.53 | 330.88 | **Down** |
| Csa19g001360 | Oleosin 5 | -1.60 | 1.48E-03 | 653.08 | 409.03 | **Down** |
| Csa11g057650 | Oleosin 2 | -1.73 | 3.66E-03 | 554.23 | 319.47 | **Down** |
| Csa11g043000 | gamma-interferon-inducible-lysosomal thiol reductase-like | -1.71 | 2.71E-03 | 481.49 | 281.14 | **Down** |
| Csa07g016060 | cupin family | -1.68 | 2.30E-03 | 323.72 | 193.07 | **Down** |
| Csa01g038030 | glycine-rich cell wall structural 2 | -53.14 | 6.56E-03 | 286.62 | 5.39 | **Down** |
| Csa12g021990 | cruciferin 3 | -3.44 | 4.31E-04 | 1851.77 | 537.88 | **Down** |
| Csa05g012650 | em GEA6 | -15.65 | 6.57E-06 | 111.46 | 7.12 | **Down** |
| Csa14g064650 | kDa class III heat shock -like | -1.56 | 5.53E-03 | 86.80 | 55.76 | **Down** |
| Csa08g062980 | PEBP (phosphatidylethanolamine-binding ) family | -1.83 | 4.38E-03 | 77.63 | 42.37 | **Down** |
| Csa09g061300 | late embryogenesis abundant | -1.96 | 2.67E-02 | 73.05 | 37.33 | **Down** |
| Csa20g001350 | aldose reductase-like | -1.53 | 1.40E-02 | 66.77 | 43.78 | **Down** |
| Csa18g035930 | HVA22 b | -1.69 | 1.91E-02 | 49.18 | 29.08 | **Down** |
| Csa08g057710 | 18 kDa seed maturation -like | -1.67 | 5.43E-03 | 45.07 | 27.02 | **Down** |
| Fold change values shown in negatives indicate decreased levels of gene expression in Camelina transgenics compared to the WT plants. DEGs, differentially expressed genes. | | | | | | |

| Table S12. List of selected DEGs showing ≥ 1.5-fold changes in expression between Camelina GPD1 transgenics and WT plants. | | | | | | |
| --- | --- | --- | --- | --- | --- | --- |
| Gene ID | **Gene description** | **Fold change** | **P-value** | **RPKMs** | | **Regulation** |
| DEGs in GPD1 *vs* WT | | | | | | |
|  |  |  |  | **WT** | **GPD1** |  |
| Csa18g033140 | gibberellin-regulated family | 1.63 | 1.65E-02 | 606.99 | 990.73 | **Up** |
| Csa10g017850 | peptide methionine sulfoxide reductase chloroplastic-like | 2.36 | 3.33E-04 | 93.61 | 220.80 | **Up** |
| Csa09g079870 | isoflavone reductase homolog P3-like | 5.59 | 2.16E-05 | 34.73 | 194.07 | **Up** |
| Csa11g019470 | ---NA--- | 2.05 | 1.08E-04 | 78.81 | 161.85 | **Up** |
| Csa12g028100 | peptide methionine sulfoxide reductase chloroplastic-like | 2.25 | 9.37E-05 | 71.69 | 161.11 | **Up** |
| Csa05g034910 | tropinone reductase homolog At1g07440-like | 1.52 | 8.73E-03 | 67.58 | 102.50 | **Up** |
| Csa08g048840 | peptide methionine sulfoxide reductase B5 | 2.33 | 1.87E-04 | 31.91 | 74.33 | **Up** |
| Csa11g077010 | SULFUR DEFICIENCY-INDUCED 1-like | 2.21 | 1.02E-02 | 27.66 | 61.15 | **Up** |
| Csa18g021440 | PREDICTED: uncharacterized protein LOC104761060 isoform X2 | 3.07 | 1.27E-03 | 19.15 | 58.72 | **Up** |
| Csa16g053900 | 5 -adenylylsulfate reductase chloroplastic | 1.80 | 3.91E-03 | 26.53 | 47.85 | **Up** |
| Csa11g059050 | cystinosin homolog | 1.59 | 7.07E-04 | 27.59 | 43.82 | **Up** |
| Csa09g075800 | kunitz-type serine protease inhibitor -like | 32.46 | 2.37E-03 | 1.30 | 42.30 | **Up** |
| Csa19g058270 | acireductone dioxygenase 3 | 1.84 | 5.34E-03 | 19.99 | 36.88 | **Up** |
| Csa05g006880 | defensin 4 | 2.05 | 3.90E-04 | 440.01 | 900.25 | **Up** |
| Csa11g017010 | 2S seed storage 2-like | 2.06 | 1.96E-04 | 228.46 | 471.17 | **Up** |
| Csa09g086190 | seed maturation | 1.54 | 4.23E-02 | 88.29 | 135.68 | **Up** |
| Csa01g035680 | gibberellin-regulated 7-like | 1.58 | 5.46E-04 | 52.46 | 83.00 | **Up** |
| Csa15g024360 | phosphoethanolamine N-methyltransferase 1-like | 1.55 | 1.69E-03 | 32.25 | 49.87 | **Up** |
| Csa11g070590 | 12S seed storage | -1.72 | 1.23E-03 | 5890.48 | 3423.36 | **Down** |
| Csa12g024730 | 2S seed storage | -1.65 | 7.06E-03 | 746.68 | 452.03 | **Down** |
| Csa10g035950 | gamma-interferon-inducible-lysosomal thiol reductase-like | -2.23 | 1.13E-03 | 515.09 | 230.53 | **Down** |
| Csa01g038030 | glycine-rich cell wall structural 2 | -11.13 | 8.36E-03 | 284.92 | 25.59 | **Down** |
| Csa04g015780 | oleosin kDa-like | -2.75 | 2.48E-02 | 240.97 | 87.76 | **Down** |
| Csa19g023490 | translationally-controlled tumor homolog | -15.09 | 1.37E-04 | 221.85 | 14.70 | **Down** |
| Csa12g021990 | cruciferin 3 | -1.84 | 6.16E-03 | 1943.54 | 1056.56 | **Down** |
| Csa19g026450 | 1-aminocyclopropane-1-carboxylate oxidase 5-like | -146.35 | 1.18E-05 | 570.44 | 3.90 | **Down** |
| Csa19g023490 | translationally-controlled tumor homolog | -462.46 | 9.55E-05 | 288.02 | 0.62 | **Down** |
| Csa15g058410 | glycine-rich cell wall structural -like | -5.38 | 1.58E-02 | 129.08 | 23.99 | **Down** |
| Csa05g012650 | em GEA6 | -4.06 | 1.91E-05 | 112.59 | 27.71 | **Down** |
| Csa03g033090 | chlorophyll a-b binding chloroplastic | -1.81 | 3.65E-03 | 86.60 | 47.97 | **Down** |
| Csa05g020850 | photosystem II type I chlorophyll a b binding | -1.54 | 1.50E-02 | 71.88 | 46.75 | **Down** |
| Csa19g025230 | methyl transferase | -146.52 | 4.87E-05 | 66.18 | 0.45 | **Down** |
| Csa19g024120 | glutamic acid-rich -like | -15.95 | 1.08E-03 | 57.47 | 3.60 | **Down** |
| Csa18g008740 | neurofilament heavy polypeptide-like | -1.68 | 9.74E-04 | 46.87 | 27.93 | **Down** |
| Csa12g069790 | glycine-rich cell wall structural -like isoform X5 | -4.99 | 2.69E-02 | 40.36 | 8.09 | **Down** |
| Fold change values shown in negatives indicate decreased levels of gene expression in Camelina transgenics compared to the WT plants. DEGs, differentially expressed genes. | | | | | | |

| Table S13. List of selected lipid-related genes differentially expressed in seeds of Camelina transgenic lines relative to WT. | | | | | |
| --- | --- | --- | --- | --- | --- |
| Gene ID | **Gene description** | **Fold change** | **P-value** | **RPKMs** | |
| Up-regulated in DGAT1 #2 | | | | | |
|  |  |  |  | **WT** | **DGAT1 #2** |
| Csa12g026280 | glycerol-3-phosphatase 1 | 1.38 | 4.99E-03 | 1.00 | 1.54 |
| Csa16g038920 | lysophosphatidyl acyltransferase 4 | 1.35 | 1.85E-02 | 0.81 | 1.24 |
| Csa14g001560 | glycerol-3-phosphate acyltransferase 4 | 1.31 | 1.82E-03 | 2.83 | 4.86 |
| Csa12g034280 | Phosphatidic acid phosphatase (PAP2) family protein | 1.31 | 2.74E-02 | 0.93 | 1.31 |
| Csa15g010030 | lipid transfer protein 6 | 1.29 | 1.53E-02 | 4.11 | 7.24 |
| Csa19g005650 | non-specific phospholipase C4 | 1.22 | 2.30E-02 | 0.76 | 1.00 |
| Csa02g059310 | Pyruvate kinase family protein | 1.21 | 3.49E-02 | 2.28 | 3.25 |
| Csa17g007350 | 3-ketoacyl-CoA synthase 2 | 1.20 | 1.92E-02 | 2.99 | 4.27 |
| Csa19g056370 | membrane bound O-acyl transferase (MBOAT) family protein | 1.17 | 7.39E-04 | 13.51 | 21.98 |
| Csa05g013960 | phosphatidylglycerolphosphate synthase 1 | 1.16 | 3.56E-02 | 3.06 | 4.10 |
| Csa03g055310 | long-chain acyl-CoA synthetase 2 | 1.12 | 1.76E-02 | 4.50 | 5.72 |
| Csa19g033960 | Cyclopropane-fatty-acyl-phospholipid synthase | 1.11 | 1.10E-02 | 8.56 | 11.07 |
| Csa08g059940 | 3-ketoacyl-CoA synthase 19 | 1.11 | 8.30E-03 | 3.50 | 4.29 |
| Csa13g011020 | phosphatidylinositol 4-OH kinase beta2 | 1.10 | 3.49E-02 | 1.35 | 1.52 |
| Csa16g027660 | beta-ketoacyl reductase 1 | 1.09 | 1.41E-02 | 16.66 | 21.72 |
| Csa17g028820 | phosphatidylinositol-4-phosphate 5-kinase 1 | 1.09 | 2.10E-02 | 12.20 | 15.41 |
| Csa09g086500 | Lipase/lipooxygenase, PLAT/LH2 family protein | 1.09 | 4.38E-02 | 6.21 | 7.67 |
| Csa19g040900 | Phospholipase A2 family protein | 1.08 | 2.90E-02 | 23.55 | 29.79 |
| Csa14g066150 | acyl-activating enzyme 18 | 1.08 | 4.23E-02 | 6.18 | 7.36 |
| Csa14g049770 | triacylglycerol lipase-like 1 | 1.07 | 2.36E-02 | 102.23 | 137.21 |
| Csa17g039510 | GDSL-like Lipase/Acylhydrolase superfamily protein | 1.07 | 2.33E-03 | 16.91 | 21.21 |
| Csa02g065010 | lipid transfer protein 4 | 1.06 | 1.04E-02 | 680.32 | 903.41 |
| Csa11g064070 | mitochondrial acyl carrier protein 3 | 1.05 | 1.69E-02 | 15.48 | 17.93 |
| Csa16g040270 | enoyl-CoA hydratase 2 | 1.05 | 3.29E-02 | 10.17 | 11.77 |
| Csa19g010510 | acyl-CoA dehydrogenase-related | 1.05 | 1.38E-02 | 7.67 | 8.60 |
| Csa10g043160 | Bifunctional inhibitor/lipid-transfer protein/seed storage 2S albumin superfamily protein | 1.04 | 4.90E-02 | 1341.25 | 1587.17 |
| Csa10g049570 | HXXXD-type acyl-transferase family protein | 1.04 | 2.90E-02 | 52.18 | 59.18 |
| Csa11g065710 | 3-ketoacyl-acyl carrier protein synthase I | 1.04 | 4.61E-02 | 37.41 | 42.04 |
| Csa06g017130 | fatty acid reductase 4 | 1.04 | 1.05E-02 | 36.87 | 41.77 |
| Csa17g016850 | MBOAT (membrane bound O-acyl transferase) family protein | 1.04 | 1.32E-02 | 6.32 | 6.89 |
| Csa14g026690 | Phosphoenolpyruvate carboxylase family protein | 1.04 | 4.88E-02 | 4.68 | 5.05 |
| Csa05g005670 | mitochondrial acyl carrier protein 1 | 1.03 | 2.52E-02 | 58.90 | 63.37 |
| Csa16g008170 | peroxisomal 3-ketoacyl-CoA thiolase 3 | 1.03 | 4.38E-02 | 43.19 | 47.22 |
| Csa06g005440 | 2-oxoacid dehydrogenases acyltransferase family protein | 1.03 | 3.88E-02 | 40.86 | 46.44 |
| Csa07g023540 | acyl carrier protein 3 | 1.02 | 4.75E-02 | 194.51 | 214.97 |
| Csa19g004750 | Plant stearoyl-acyl-carrier-protein desaturase family protein | 1.02 | 4.13E-03 | 67.58 | 70.85 |
| Csa12g079300 | NAD-dependent glycerol-3-phosphate dehydrogenase family protein | 1.02 | 4.00E-02 | 33.89 | 35.54 |
| Down-regulated in DGAT1 #2 | | | | | |
|  |  |  |  | **WT** | **DGAT1 #2** |
| Csa15g044570 | Cyclopropane-fatty-acyl-phospholipid synthase | -18.24 | 3.91E-03 | 0.89 | 0.03 |
| Csa06g017130 | fatty acid reductase 4 | -4.48 | 3.49E-03 | 5.31 | 0.48 |
| Csa06g040850 | glycerol-3-phosphate acyltransferase 6 | -1.46 | 2.87E-02 | 0.97 | 0.55 |
| Csa19g013410 | phosphatidyl inositol monophosphate 5 kinase | -1.37 | 2.98E-02 | 0.59 | 0.37 |
| Csa04g015780 | oleosin 4 | -1.36 | 1.57E-02 | 214.47 | 51.81 |
| Csa18g042120 | Phosphatidic acid phosphatase (PAP2) family protein | -1.35 | 3.24E-02 | 0.97 | 0.61 |
| Csa03g055310 | long-chain acyl-CoA synthetase 2 | -1.34 | 3.77E-03 | 2.46 | 1.48 |
| Csa19g042120 | Bifunctional inhibitor/lipid-transfer protein/seed storage 2S albumin superfamily protein | -1.22 | 3.61E-02 | 9.67 | 5.99 |
| Csa15g072100 | 3-ketoacyl-CoA synthase 9 | -1.20 | 4.89E-02 | 3.61 | 2.56 |
| Csa10g007610 | 3-ketoacyl-CoA synthase 18 | -1.19 | 8.51E-03 | 11.83 | 7.60 |
| Csa08g008530 | acyl activating enzyme 5 | -1.19 | 1.99E-02 | 1.69 | 1.32 |
| Csa05g011450 | phosphatidylinositol- 4-phosphate 5-kinase 5 | -1.19 | 1.03E-02 | 1.49 | 1.10 |
| Csa08g062980 | PEBP (phosphatidylethanolamine-binding protein) family protein | -1.17 | 3.33E-03 | 70.09 | 36.41 |
| Csa16g014970 | fatty acid desaturase 3 | -1.15 | 3.59E-02 | 75.88 | 43.51 |
| Csa18g010110 | 3-ketoacyl-CoA synthase 20 | -1.15 | 3.42E-02 | 3.22 | 2.47 |
| Csa06g052640 | Fatty acid/sphingolipid desaturase | -1.14 | 4.58E-02 | 6.34 | 4.77 |
| Csa15g002280 | lipid phosphate phosphatase 3 | -1.14 | 2.90E-02 | 2.85 | 2.21 |
| Csa04g024240 | fatty acid reductase 5 | -1.13 | 1.18E-02 | 21.16 | 14.75 |
| Csa20g036130 | fatty acid reductase 1 | -1.13 | 8.53E-03 | 1.19 | 0.95 |
| Csa01g018440 | phosphatidic acid phosphatase-related / PAP2-related | -1.12 | 4.68E-02 | 4.90 | 3.84 |
| Csa10g024840 | Long-chain fatty alcohol dehydrogenase family protein | -1.12 | 1.05E-02 | 3.87 | 3.08 |
| Csa20g037970 | acyl-activating enzyme 17 | -1.10 | 2.60E-02 | 35.75 | 24.99 |
| Csa09g035780 | Lecithin:cholesterol acyltransferase family protein | -1.10 | 2.27E-02 | 14.16 | 10.97 |
| Csa02g033710 | peroxisomal 3-keto-acyl-CoA thiolase 2 | -1.10 | 4.16E-02 | 9.52 | 7.49 |
| Csa06g024860 | esterase/lipase/thioesterase family protein | -1.10 | 3.82E-02 | 5.09 | 4.12 |
| Csa12g011550 | 1-phosphatidylinositol-4-phosphate 5-kinases | -1.10 | 3.61E-02 | 3.40 | 2.83 |
| Csa12g002610 | Lipase/lipooxygenase, PLAT/LH2 family protein | -1.09 | 4.69E-02 | 22.28 | 17.10 |
| Csa19g028170 | trigalactosyldiacylglycerol2 | -1.09 | 1.45E-02 | 5.83 | 4.86 |
| Csa17g092850 | lysophospholipase 2 | -1.09 | 8.71E-03 | 5.40 | 4.54 |
| Csa01g003020 | lecithin:cholesterol acyltransferase 3 | -1.08 | 1.58E-02 | 5.80 | 4.88 |
| Csa12g036760 | Diacylglycerol kinase family protein | -1.07 | 1.66E-02 | 8.45 | 7.13 |
| Csa16g047830 | 3-ketoacyl-CoA synthase 10 | -1.07 | 3.29E-02 | 7.21 | 6.20 |
| Csa17g009720 | acyl-CoA oxidase 3 | -1.07 | 1.45E-02 | 5.05 | 4.37 |
| Csa01g013220 | fatty acid desaturase 2 | -1.06 | 4.02E-02 | 221.46 | 161.02 |
| Csa15g052810 | fatA acyl-ACP thioesterase | -1.06 | 2.27E-02 | 39.31 | 32.50 |
| Csa10g049100 | NAD-dependent glycerol-3-phosphate dehydrogenase family protein | -1.06 | 4.38E-02 | 27.16 | 22.31 |
| Csa03g060560 | phosphoenolpyruvate carboxylase 1 | -1.06 | 4.56E-02 | 7.64 | 6.68 |
| Csa11g014490 | Phospholipase A2 family protein | -1.05 | 3.13E-02 | 13.68 | 12.10 |
| Csa14g016600 | Phosphatidylinositol 3- and 4-kinase family protein | -1.05 | 1.30E-02 | 9.60 | 8.41 |
| Csa17g002220 | Acyl-CoA thioesterase family protein | -1.05 | 3.29E-02 | 5.11 | 4.64 |
| Csa11g006770 | Arabidopsis phospholipase-like protein (PEARLI 4) family | -1.05 | 4.53E-02 | 4.91 | 4.41 |
| Csa07g033170 | beta-ketoacyl reductase 1 | -1.04 | 3.12E-02 | 24.43 | 21.81 |
| Csa12g007640 | phospholipase D delta | -1.04 | 2.43E-02 | 11.61 | 10.51 |
| Csa04g052350 | lipid transfer protein 1 | -1.03 | 2.42E-02 | 235.35 | 193.75 |
| Csa11g085290 | acyl-CoA binding protein 1 | -1.03 | 3.94E-02 | 12.24 | 11.11 |
| Csa12g028090 | oleosin 1 | -1.02 | 1.93E-02 | 2945.32 | 2609.80 |
| Csa11g057650 | oleosin 2 | -1.02 | 4.86E-02 | 1247.51 | 1005.42 |
| Csa11g084730 | plastidic pyruvate kinase beta subunit 1 | -1.02 | 2.50E-02 | 120.16 | 111.02 |
| Csa05g006640 | Plant stearoyl-acyl-carrier-protein desaturase family protein | -1.02 | 1.88E-02 | 86.14 | 78.60 |
| Csa10g042420 | Oleosin family protein | -1.01 | 1.26E-02 | 2355.26 | 2221.56 |
| Csa12g022880 | acyl-CoA binding protein 2 | -1.01 | 1.14E-02 | 24.67 | 23.32 |
| Csa13g010700 | Pyruvate kinase family protein | -1.01 | 9.64E-03 | 19.78 | 19.16 |
| Up-regulated in GPD1 #2 | | | | | |
|  |  |  |  | **WT** | **GPD1 #2** |
| Csa02g065020 | lipid transfer protein 4 | 2.14 | 4.90E-02 | 1.46 | 5.42 |
| Csa15g019360 | phosphoenolpyruvate carboxylase 3 | 1.94 | 4.93E-02 | 0.30 | 0.64 |
| Csa19g012130 | lipid transfer protein 6 | 1.73 | 3.89E-02 | 0.64 | 1.26 |
| Csa11g092400 | phospholipase C1 | 1.67 | 2.74E-02 | 0.34 | 0.59 |
| Csa01622s010 | acyl-activating enzyme 17 | 1.63 | 1.12E-02 | 2.88 | 7.83 |
| Csa18g024410 | O-acyltransferase (WSD1-like) family protein | 1.62 | 7.52E-03 | 1.06 | 2.09 |
| Csa01g021830 | lysophosphatidyl acyltransferase 5 | 1.62 | 4.34E-03 | 0.48 | 0.82 |
| Csa14g014970 | Bifunctional inhibitor/lipid-transfer protein/seed storage 2S albumin superfamily protein | 1.56 | 4.16E-02 | 1.14 | 2.17 |
| Csa17g007110 | phospholipid sterol acyl transferase 1 | 1.55 | 1.56E-02 | 0.34 | 0.54 |
| Csa03g009940 | glycerol-3-phosphate acyltransferase 1 | 1.52 | 3.71E-02 | 0.81 | 1.40 |
| Csa00441s260 | phospholipase A2-beta | 1.41 | 9.68E-03 | 0.79 | 1.18 |
| Csa18g030890 | Fatty acid hydroxylase superfamily | 1.38 | 4.12E-02 | 0.44 | 0.62 |
| Csa13g049040 | phospholipase D gamma 1 | 1.37 | 1.76E-03 | 1.08 | 1.63 |
| Csa12g011830 | sulfoquinovosyldiacylglycerol 1 | 1.33 | 2.32E-02 | 2.74 | 4.54 |
| Csa02g059310 | Pyruvate kinase family protein | 1.23 | 1.63E-02 | 2.28 | 3.11 |
| Csa16g052740 | Phosphatidylinositol-4-phosphate 5-kinase family protein | 1.20 | 3.26E-02 | 2.69 | 3.67 |
| Csa09g069120 | fatty acid reductase 6 | 1.19 | 1.02E-02 | 9.11 | 13.80 |
| Csa13g025900 | fatty acid reductase 7 | 1.17 | 3.31E-02 | 2.68 | 3.42 |
| Csa19g004730 | lipid phosphate phosphatase 3 | 1.17 | 2.78E-02 | 1.68 | 2.05 |
| Csa09g034350 | fatty acid reductase 4 | 1.16 | 2.80E-02 | 11.61 | 16.86 |
| Csa07g017780 | Lipases | 1.16 | 1.48E-02 | 1.92 | 2.36 |
| Csa17g002080 | glycerol-3-phosphate acyltransferase 4 | 1.15 | 3.04E-02 | 2.10 | 2.57 |
| Csa14g055640 | long-chain acyl-CoA synthetase 2 | 1.14 | 3.95E-02 | 6.88 | 9.03 |
| Csa15g044570 | Cyclopropane-fatty-acyl-phospholipid synthase | 1.13 | 1.46E-02 | 7.37 | 9.52 |
| Csa17g007350 | 3-ketoacyl-CoA synthase 2 | 1.13 | 4.30E-02 | 2.99 | 3.56 |
| Csa16g003620 | Fatty acid/sphingolipid desaturase | 1.13 | 1.65E-02 | 1.31 | 1.50 |
| Csa14g007750 | Fatty acid desaturase family protein | 1.10 | 3.71E-02 | 14.04 | 18.47 |
| Csa13g009890 | diacylglycerol kinase1 | 1.10 | 4.05E-02 | 4.78 | 5.60 |
| Csa17g093650 | phosphoenolpyruvate carboxylase 1 | 1.09 | 3.91E-02 | 9.79 | 11.69 |
| Csa16g043920 | Lipase/lipooxygenase, PLAT/LH2 family protein | 1.09 | 4.92E-02 | 8.33 | 10.14 |
| Csa14g066150 | acyl-activating enzyme 18 | 1.09 | 3.16E-02 | 6.18 | 7.35 |
| Csa10g020840 | cytidinediphosphate diacylglycerol synthase 2 | 1.09 | 2.15E-02 | 3.82 | 4.32 |
| Csa09g050690 | non-specific phospholipase C6 | 1.09 | 1.02E-02 | 2.99 | 3.39 |
| Csa11g088060 | pyruvate decarboxylase-2 | 1.08 | 2.44E-02 | 7.74 | 8.92 |
| Csa10g022650 | fatty acid hydroxylase 2 | 1.08 | 4.74E-02 | 6.03 | 7.08 |
| Csa16g055340 | 3-ketoacyl-acyl carrier protein synthase III | 1.07 | 4.68E-02 | 19.46 | 22.64 |
| Csa12g077540 | HXXXD-type acyl-transferase family protein | 1.07 | 2.10E-02 | 15.76 | 19.31 |
| Csa03g011960 | fatty acyl-ACP thioesterases B | 1.07 | 1.95E-03 | 7.26 | 8.04 |
| Csa17g070600 | Plant stearoyl-acyl-carrier-protein desaturase family protein | 1.06 | 4.78E-02 | 72.66 | 93.82 |
| Csa06g041400 | lipid transfer protein 1 | 1.06 | 2.43E-02 | 74.47 | 89.17 |
| Csa17g028820 | phosphatidylinositol-4-phosphate 5-kinase 1 | 1.06 | 3.94E-02 | 12.20 | 13.63 |
| Csa17g025060 | fatty acid biosynthesis 1 | 1.06 | 1.98E-02 | 11.83 | 13.23 |
| Csa16g040270 | enoyl-CoA hydratase 2 | 1.06 | 3.80E-02 | 10.17 | 11.74 |
| Csa16g017490 | aminophospholipid ATPase 3 | 1.06 | 1.80E-02 | 3.87 | 4.16 |
| Csa16g027640 | beta-ketoacyl reductase 1 | 1.05 | 2.38E-02 | 27.34 | 30.58 |
| Csa19g033920 | fatty alcohol oxidase 3 | 1.05 | 4.62E-02 | 11.74 | 13.34 |
| Csa09g097070 | CDP-diacylglycerol synthase 1 | 1.05 | 4.61E-02 | 5.22 | 5.66 |
| Csa14g049770 | triacylglycerol lipase-like 1 | 1.04 | 4.76E-02 | 102.23 | 123.16 |
| Csa17g050040 | acyl-CoA-binding protein 6 | 1.04 | 2.07E-02 | 81.59 | 89.58 |
| Csa05g059570 | acyl carrier protein 3 | 1.04 | 2.73E-03 | 70.07 | 83.36 |
| Csa11g058960 | NAD-dependent glycerol-3-phosphate dehydrogenase family protein | 1.04 | 8.42E-03 | 26.61 | 28.20 |
| Csa09g087750 | spermidine disinapoyl acyltransferase | 1.04 | 5.00E-02 | 20.69 | 21.95 |
| Csa11g032960 | acyl-CoA oxidase 1 | 1.04 | 2.40E-02 | 11.30 | 12.04 |
| Csa11g003650 | phosphoenolpyruvate carboxykinase 1 | 1.03 | 2.69E-02 | 46.34 | 48.57 |
| Csa17g092830 | lysophospholipase 2 | 1.01 | 2.94E-02 | 152.67 | 163.21 |
| Down-regulated in GPD1 #2 | | | | | |
|  |  |  |  | **WT** | **GPD1 #2** |
| Csa04g024230 | fatty acid reductase 4 | -41.82 | 1.36E-05 | 4.55 | 0.04 |
| Csa08g062980 | PEBP (phosphatidylethanolamine-binding protein) family protein | -17.43 | 2.31E-03 | 2.79 | 0.07 |
| Csa19g023650 | phospholipase D P1 | -7.43 | 1.27E-02 | 2.17 | 0.16 |
| Csa15g044570 | Cyclopropane-fatty-acyl-phospholipid synthase | -7.17 | 1.76E-03 | 0.89 | 0.08 |
| Csa04g012540 | Esterase/lipase/thioesterase family protein | -4.26 | 1.09E-03 | 1.29 | 0.20 |
| Csa01g003220 | non-specific phospholipase C4 | -2.68 | 1.41E-02 | 0.78 | 0.22 |
| Csa01g021440 | Oleosin family protein | -2.47 | 7.20E-03 | 3.86 | 0.85 |
| Csa01g021080 | Bifunctional inhibitor/lipid-transfer protein/seed storage 2S albumin superfamily protein | -2.37 | 1.89E-02 | 0.98 | 0.31 |
| Csa04g051790 | glycerol-3-phosphate acyltransferase 6 | -1.97 | 2.29E-02 | 0.62 | 0.25 |
| Csa16g028550 | 3-ketoacyl-CoA synthase 6 | -1.66 | 3.48E-02 | 1.04 | 0.51 |
| Csa15g015170 | glycerol-3-phosphate acyltransferase 5 | -1.57 | 1.08E-02 | 1.55 | 0.78 |
| Csa15g002250 | Fatty acid hydroxylase superfamily protein | -1.38 | 2.34E-02 | 19.48 | 7.49 |
| Csa09g034370 | fatty acid reductase 5 | -1.35 | 2.63E-02 | 7.89 | 3.96 |
| Csa18g026810 | MBOAT (membrane bound O-acyl transferase) family protein | -1.28 | 4.13E-02 | 0.95 | 0.64 |
| Csa06g008780 | oleosin 4 | -1.25 | 3.81E-02 | 191.30 | 64.84 |
| Csa10g028850 | phosphatidylserine decarboxylase 1 | -1.25 | 1.43E-03 | 1.20 | 0.83 |
| Csa05g083360 | hydroxypyruvate reductase | -1.23 | 2.78E-02 | 4.47 | 2.84 |
| Csa13g019170 | acyl activating enzyme 5 | -1.16 | 4.36E-02 | 0.82 | 0.63 |
| Csa08g017410 | acyl-activating enzyme 17 | -1.13 | 1.41E-03 | 19.23 | 12.62 |
| Csa09g035780 | Lecithin:cholesterol acyltransferase family protein | -1.13 | 2.66E-03 | 14.16 | 10.01 |
| Csa16g014970 | fatty acid desaturase 3 | -1.12 | 4.89E-02 | 75.88 | 46.71 |
| Csa10g007610 | 3-ketoacyl-CoA synthase 18 | -1.12 | 5.54E-03 | 11.83 | 8.69 |
| Csa14g007700 | acyl-CoA oxidase 3 | -1.11 | 2.58E-02 | 4.30 | 3.31 |
| Csa19g015230 | fatty acid desaturase 7 | -1.11 | 4.47E-02 | 2.41 | 1.92 |
| Csa11g002750 | Lipase/lipooxygenase, PLAT/LH2 family protein | -1.10 | 3.72E-02 | 22.75 | 16.68 |
| Csa11g015010 | O-acyltransferase (WSD1-like) family protein | -1.10 | 2.47E-02 | 5.71 | 4.51 |
| Csa15g020490 | fatty acid desaturase 5 | -1.09 | 3.14E-02 | 10.33 | 7.90 |
| Csa06g052640 | Fatty acid/sphingolipid desaturase | -1.09 | 2.86E-02 | 6.34 | 4.92 |
| Csa11g020530 | acyl-CoA-binding domain 3 | -1.08 | 2.49E-02 | 6.05 | 5.03 |
| Csa01g020470 | Dihydroxyacetone kinase | -1.08 | 3.76E-02 | 5.67 | 4.70 |
| Csa11g057650 | oleosin 2 | -1.07 | 3.20E-02 | 347.98 | 237.69 |
| Csa18g033950 | glycerol-3-phosphate acyltransferase 9 | -1.05 | 4.80E-02 | 13.83 | 11.40 |
| Csa12g007640 | phospholipase D delta | -1.04 | 1.16E-02 | 11.61 | 10.35 |
| Csa03g059760 | lysophospholipase 2 | -1.03 | 4.83E-02 | 40.97 | 36.08 |
| Csa16g017420 | pyruvate dehydrogenase complex E1 alpha subunit | -1.02 | 2.77E-02 | 21.64 | 18.99 |

| Table S14. List of selected genes encode transcription factors which are differentially expressed in seeds of Camelina transgenic lines relative to WT. | | | | | | |
| --- | --- | --- | --- | --- | --- | --- |
| Gene ID | **Gene description** | **Fold change** | **P-value** | **RPKMs** | | **Regulation** |
| Up-regulated in DGAT1m | | | | | | |
| Csa19g013530 | sequence-specific DNA binding transcription factor | 1.59 | 9.60E-03 | 4.84 | 7.70 | UP |
| Csa14g027760 | AGAMOUS-like 87 | 1.87 | 2.26E-03 | 2.66 | 4.97 | UP |
| Csa20g071260 | ethylene-responsive transcription factor 9-like | 1.65 | 1.25E-02 | 1.39 | 2.29 | UP |
| Csa17g039230 | ethylene-responsive transcription factor 11-like | 1.73 | 6.86E-03 | 0.76 | 1.31 | UP |
| Csa20g010010 | ethylene-responsive transcription factor ERF106 | 1.99 | 2.94E-03 | 0.46 | 0.91 | UP |
| Csa11g066870 | dnaJ homolog subfamily C member 2-like | 1.64 | 7.18E-03 | 0.52 | 0.85 | UP |
| Csa04g051940 | trihelix transcription factor GT-3b-like | 1.52 | 2.64E-02 | 0.50 | 0.76 | UP |
| Csa01g033020 | transcription factor ABORTED MICROSPORES | 1.71 | 2.86E-02 | 0.35 | 0.60 | UP |
| Csa07g063560 | WRKY transcription factor 6-like | 2.07 | 1.69E-02 | 0.27 | 0.55 | UP |
| Csa06g002590 | NAC domain-containing 55-like isoform X2 | 1.75 | 1.94E-03 | 0.31 | 0.54 | UP |
| Csa17g095950 | NAC domain-containing 21 22-like | 1.50 | 4.41E-02 | 1.16 | 1.74 | UP |
| Csa04g054530 | ethylene-responsive transcription factor ABI4-like | 1.54 | 1.05E-02 | 0.76 | 1.17 | UP |
| Csa17g017170 | ethylene-responsive transcription factor ERF088-like isoform X1 | 2.30 | 4.75E-02 | 0.35 | 0.80 | UP |
| Csa01g012030 | dehydration-responsive element-binding 2B-like | 2.00 | 2.47E-02 | 0.28 | 0.57 | UP |
| Csa11g003480 | dof zinc finger -like | 1.93 | 2.43E-02 | 0.29 | 0.56 | UP |
| Csa06g002590 | NAC domain-containing 55-like isoform X2 | 1.80 | 4.59E-02 | 0.30 | 0.55 | UP |
| Down-regulated in DGAT1m | | | | | | |
|  |  |  |  |  |  |  |
| Csa09g034290 | ABSCISIC ACID-INSENSITIVE 5 1 | -1.54 | 9.47E-03 | 39.65 | 25.70 | Down |
| Csa02g051710 | heat stress transcription factor A-9-like | -1.91 | 7.04E-03 | 18.21 | 9.51 | Down |
| Csa14g042020 | pinoresinol reductase 1-like | -2.16 | 6.02E-03 | 11.42 | 5.28 | Down |
| Csa17g001540 | Homeodomain-like superfamily | -1.87 | 4.10E-03 | 9.45 | 5.04 | Down |
| Csa05g085820 | Agamous-like MADS-box AGL8 isogeny | -2.87 | 9.53E-03 | 6.91 | 2.41 | Down |
| Csa17g022060 | myb domain 58 | -1.55 | 5.63E-03 | 6.80 | 4.37 | Down |
| Csa14g002120 | LHY isoform X1 | -2.03 | 8.08E-03 | 6.36 | 3.13 | Down |
| Csa14g055680 | B3 domain-containing At1g49475-like | -1.58 | 3.63E-02 | 6.21 | 3.94 | Down |
| Csa11g086010 | heat stress transcription factor A-9-like | -2.49 | 1.05E-02 | 5.71 | 2.30 | Down |
| Csa01g030490 | multi -bridging factor 1c | -1.60 | 1.08E-02 | 4.65 | 2.91 | Down |
| Csa10g004010 | heat stress transcription factor B-1-like | -1.60 | 2.31E-02 | 4.24 | 2.64 | Down |
| Csa19g036400 | multi -bridging factor 1c | -1.53 | 4.36E-02 | 4.04 | 2.64 | Down |
| Csa18g025310 | heat stress transcription factor A-9-like | -2.39 | 1.02E-02 | 3.67 | 1.53 | Down |
| Csa06g025000 | zinc-finger homeodomain 7-like | -1.93 | 1.63E-03 | 3.48 | 1.80 | Down |
| Csa14g004860 | zinc finger (CCCH-type) family | -1.99 | 2.73E-03 | 3.22 | 1.62 | Down |
| Csa09g002470 | zinc-finger homeodomain 7-like | -1.99 | 2.18E-02 | 3.00 | 1.51 | Down |
| Csa12g074610 | REVEILLE 2-like | -1.59 | 8.96E-03 | 2.98 | 1.87 | Down |
| Csa17g001720 | ethylene-responsive transcription factor ERF023-like | -1.85 | 3.40E-02 | 2.39 | 1.30 | Down |
| Csa13g007380 | dehydration-responsive element-binding 2A-like | -1.74 | 3.76E-03 | 2.31 | 1.32 | Down |
| Csa01g018090 | NAC domain-containing 55-like | -2.42 | 1.73E-02 | 2.26 | 0.93 | Down |
| Csa11g004370 | transcription factor MYB44-like | -1.64 | 2.53E-02 | 2.21 | 1.35 | Down |
| Csa04g054400 | zinc finger CCCH domain-containing 29-like | -1.54 | 9.93E-03 | 1.92 | 1.25 | Down |
| Csa18g034940 | ethylene-responsive transcription factor ERF104-like | -2.65 | 3.36E-02 | 1.86 | 0.70 | Down |
| Csa15g031170 | 2-oxoglutarate (2OG) and Fe(II)-dependent oxygenase superfamily | -2.48 | 8.81E-04 | 1.77 | 0.72 | Down |
| Csa16g031310 | homeobox-leucine zipper ATHB-13-like | -1.61 | 1.96E-02 | 1.74 | 1.08 | Down |
| Csa11g078560 | GATA transcription factor 16-like | -1.99 | 4.27E-02 | 1.50 | 0.76 | Down |
| Csa05g044630 | plant-specific transcription factor YABBY family | -1.92 | 3.29E-03 | 1.47 | 0.77 | Down |
| Csa09g080130 | dehydration-responsive element-binding 2D-like | -9.58 | 1.20E-02 | 1.43 | 0.15 | Down |
| Csa15g016690 | myb family transcription factor APL-like isoform X1 | -1.67 | 2.49E-02 | 1.43 | 0.86 | Down |
| Csa03g020380 | B3 domain-containing At1g16640-like | -2.45 | 4.99E-02 | 1.42 | 0.58 | Down |
| Csa15g013970 | NAC domain-containing 74-like | -1.64 | 9.55E-03 | 1.36 | 0.83 | Down |
| Csa09g088620 | CUP-SHAPED COTYLEDON 3-like | -2.33 | 3.76E-03 | 1.21 | 0.52 | Down |
| Csa04g009910 | AT3g25710 K13N2_1 | -1.69 | 2.47E-02 | 1.19 | 0.70 | Down |
| Csa11g005420 | SHI RELATED SEQUENCE 2-like | -1.67 | 3.67E-02 | 1.14 | 0.68 | Down |
| Csa02g055900 | AT5g54630 MRB17_13 | -1.77 | 1.01E-03 | 1.11 | 0.63 | Down |
| Csa09g058950 | heat stress transcription factor A-7a-like | -2.40 | 4.75E-02 | 1.07 | 0.44 | Down |
| Csa11g097430 | ethylene-responsive transcription factor ERF104-like | -6.20 | 1.59E-03 | 1.06 | 0.17 | Down |
| Csa14g005070 | G-box-binding factor 4-like | -2.07 | 1.60E-02 | 1.00 | 0.48 | Down |
| Csa02g068570 | ethylene-responsive transcription factor ERF104-like | -6.94 | 9.84E-04 | 0.99 | 0.14 | Down |
| Csa22397s010 | zinc finger (C2H2 type) family | -3.00 | 3.74E-02 | 0.79 | 0.27 | Down |
| Csa06g029500 | AP2-like ethylene-responsive transcription factor SMZ | -2.70 | 8.20E-03 | 0.77 | 0.29 | Down |
| Csa05g001300 | zinc finger CONSTANS-LIKE 13-like | -1.92 | 4.69E-02 | 0.65 | 0.34 | Down |
| Csa06g005270 | transcription factor AIG1-like | -2.97 | 4.70E-02 | 0.61 | 0.21 | Down |
| Csa05g017850 | ABSCISIC ACID-INSENSITIVE 5-like | -2.72 | 2.13E-02 | 0.61 | 0.22 | Down |
| Csa08g062200 | dof zinc finger -like | -1.78 | 3.93E-02 | 0.61 | 0.34 | Down |
| Csa02g035390 | GATA transcription factor 16-like | -2.64 | 3.68E-02 | 0.56 | 0.21 | Down |
| Csa04g043660 | Agamous-like MADS-box AGL16 | -1.86 | 1.69E-02 | 0.52 | 0.28 | Down |
| Up-regulated in GPD1 | | | | | | |
|  |  |  |  |  |  |  |
| Csa17g050420 | pinoresinol reductase 1-like | 1.57 | 1.79E-03 | 13.96 | 21.93 | Up |
| Csa05g094340 | phytochrome interacting factor 3-like 2 | 1.52 | 1.01E-02 | 4.33 | 6.61 | Up |
| Csa15g024960 | NAC domain-containing 100-like | 1.54 | 1.11E-02 | 2.86 | 4.42 | Up |
| Csa08g054480 | ETHYLENE INSENSITIVE 3-like 4 | 1.64 | 3.27E-02 | 2.36 | 3.86 | Up |
| Csa04g002510 | homeobox-leucine zipper ATHB-17-like isoform X1 | 1.59 | 3.11E-02 | 2.36 | 3.75 | Up |
| Csa07g053360 | probable WRKY transcription factor 15 | 1.59 | 9.15E-03 | 1.49 | 2.37 | Up |
| Csa04g063650 | AGAMOUS-like 20 | 1.56 | 7.25E-03 | 1.31 | 2.04 | Up |
| Csa05g002800 | probable WRKY transcription factor 46 | 3.38 | 9.90E-03 | 0.40 | 1.34 | Up |
| Csa03g028490 | homeobox-leucine zipper ATHB-23 | 1.63 | 1.67E-03 | 0.77 | 1.26 | Up |
| Csa15g021160 | ethylene-responsive transcription factor ERF036-like | 2.88 | 4.84E-02 | 0.37 | 1.06 | Up |
| Csa06g052800 | ethylene-responsive transcription factor CRF5 | 1.54 | 6.66E-03 | 0.64 | 0.99 | Up |
| Csa02g072750 | NAC domain-containing 78-like | 1.89 | 1.05E-02 | 0.50 | 0.94 | Up |
| Csa17g034300 | C2H2-like zinc finger | 1.72 | 4.48E-02 | 0.55 | 0.94 | Up |
| Csa18g005870 | dnaJ homolog subfamily C member 2-like | 1.97 | 2.91E-02 | 0.47 | 0.93 | Up |
| Csa04g051940 | trihelix transcription factor GT-3b-like | 1.64 | 2.08E-02 | 0.51 | 0.83 | Up |
| Csa02g004650 | NAC domain containing 69 | 2.14 | 2.29E-02 | 0.38 | 0.81 | Up |
| Csa20g010010 | ethylene-responsive transcription factor ERF106 | 1.64 | 2.19E-03 | 0.46 | 0.75 | Up |
| Csa01g033020 | basic helix-loop-helix (bHLH) DNA-binding superfamily | 2.01 | 2.45E-02 | 0.36 | 0.71 | Up |
| Csa04g051380 | zinc-finger 10 | 2.39 | 4.45E-02 | 0.29 | 0.69 | Up |
| Csa04330s010 | ethylene-responsive transcription factor CRF5- partial | 1.68 | 2.02E-02 | 0.41 | 0.68 | Up |
| Csa09g095630 | NAC domain containing 25 | 1.66 | 1.01E-03 | 0.39 | 0.65 | Up |
| Csa07g063560 | WRKY transcription factor 6-like | 2.71 | 7.04E-04 | 0.23 | 0.62 | Up |
| Csa04g030640 | transcription factor PRE4-like | 2.11 | 3.17E-02 | 0.29 | 0.61 | Up |
| Csa06g024450 | dof zinc finger -like | 1.69 | 2.73E-02 | 0.36 | 0.61 | Up |
| Csa06g002590 | NAC domain containing 35 | 1.89 | 1.37E-03 | 0.31 | 0.59 | Up |
| Csa17g033710 | Homeobox-leucine zipper family | 1.80 | 2.15E-02 | 0.31 | 0.56 | Up |
| Csa12g048260 | homeobox-leucine zipper HAT1 (hd-zip 1) | 1.94 | 2.38E-02 | 0.29 | 0.56 | Up |
| Csa09g002490 | GATA transcription factor 18-like | 1.52 | 4.84E-02 | 0.36 | 0.55 | Up |
| Down-regulated in GPD1 | | | | | | |
|  |  |  |  |  |  |  |
| Csa19g023630 | AP2 domain containing | -14.72 | 1.81E-04 | 117.74 | 8.00 | Down |
| Csa19g026420 | AP2 B3-like transcriptional factor family | -5.57 | 4.30E-03 | 18.34 | 3.30 | Down |
| Csa02g051710 | heat stress transcription factor A-9-like | -1.63 | 2.63E-02 | 18.30 | 11.23 | Down |
| Csa14g042020 | pinoresinol reductase 1-like | -1.54 | 2.57E-02 | 11.47 | 7.44 | Down |
| Csa19g023770 | GATA transcription factor 17 | -19.65 | 5.18E-03 | 11.13 | 0.57 | Down |
| Csa05g085820 | Agamous-like MADS-box AGL8 isogeny | -1.95 | 2.62E-02 | 6.95 | 3.57 | Down |
| Csa11g086010 | heat stress transcription factor A-9-like | -2.62 | 3.36E-03 | 5.70 | 2.17 | Down |
| Csa18g034930 | ethylene-responsive transcription factor ERF107-like | -1.88 | 3.77E-02 | 5.57 | 2.96 | Down |
| Csa11g004520 | heat stress transcription factor B-1-like | -1.69 | 3.43E-02 | 4.89 | 2.90 | Down |
| Csa20g024330 | zinc finger (C2H2 type) family | -1.72 | 1.92E-02 | 4.36 | 2.54 | Down |
| Csa10g004010 | heat stress transcription factor B-1-like | -1.65 | 1.11E-02 | 4.27 | 2.59 | Down |
| Csa14g004860 | zinc finger (CCCH-type) family | -1.78 | 2.79E-03 | 3.21 | 1.81 | Down |
| Csa11g041340 | pinoresinol reductase 2 | -1.55 | 8.31E-03 | 2.86 | 1.85 | Down |
| Csa11g089590 | probable WRKY transcription factor 2 | -1.51 | 1.46E-02 | 2.72 | 1.80 | Down |
| Csa07g053370 | ethylene-responsive transcription factor ERF008-like | -1.67 | 1.44E-02 | 2.51 | 1.51 | Down |
| Csa01g018090 | NAC domain-containing 55-like | -2.23 | 1.38E-02 | 2.27 | 1.02 | Down |
| Csa03g001950 | ethylene-responsive transcription factor ERF023-like | -1.60 | 1.79E-02 | 2.13 | 1.33 | Down |
| Csa05g088940 | myb-like transcription factor family | -1.74 | 1.09E-05 | 1.99 | 1.14 | Down |
| Csa18g034940 | ethylene-responsive transcription factor ERF104-like | -3.01 | 2.00E-02 | 1.85 | 0.61 | Down |
| Csa15g031170 | 2-oxoglutarate (2OG) and Fe(II)-dependent oxygenase superfamily | -2.15 | 1.38E-03 | 1.76 | 0.82 | Down |
| Csa02g067810 | dof zinc finger -like | -1.73 | 2.69E-02 | 1.58 | 0.91 | Down |
| Csa19g022270 | NAC domain-containing 55-like | -6.54 | 3.46E-03 | 1.57 | 0.24 | Down |
| Csa15g020040 | NAC domain-containing 55-like | -4.56 | 8.58E-03 | 1.52 | 0.33 | Down |
| Csa02g021670 | HMG (high mobility group) box | -1.59 | 4.54E-02 | 1.45 | 0.91 | Down |
| Csa09g080130 | dehydration-responsive element-binding 2D-like | -9.39 | 1.22E-02 | 1.44 | 0.15 | Down |
| Csa20g049180 | GATA transcription factor 12-like | -1.62 | 4.02E-02 | 1.44 | 0.89 | Down |
| Csa09g088620 | CUP-SHAPED COTYLEDON 3-like | -1.67 | 1.74E-02 | 1.22 | 0.73 | Down |
| Csa15g071680 | C2H2-like zinc finger | -1.71 | 2.72E-02 | 1.14 | 0.66 | Down |
| Csa11g005420 | SHI RELATED SEQUENCE 2-like | -1.70 | 0.016442 | 1.12 | 0.66 | Down |
| Csa02g055900 | zinc finger | -1.51 | 0.011396 | 1.10 | 0.72 | Down |
| Csa09g058950 | heat stress transcription factor A-7a-like | -2.70 | 3.37E-02 | 1.06 | 0.39 | Down |
| Csa11g097430 | ethylene-responsive transcription factor ERF104-like | -3.86 | 4.80E-04 | 1.06 | 0.27 | Down |
| Csa02g068570 | ethylene-responsive transcription factor ERF104-like | -4.75 | 8.07E-04 | 0.98 | 0.21 | Down |
| Csa34454s010 | DOF zinc finger -like | -7.58 | 2.58E-02 | 0.95 | 0.13 | Down |
| Csa06g029500 | AP2-like ethylene-responsive transcription factor SMZ | -1.90 | 9.68E-03 | 0.78 | 0.41 | Down |
| Csa19g024040 | transcription factor bHLH147-like | -7.15 | 3.95E-02 | 0.77 | 0.11 | Down |
| Csa18g025650 | C2H2 and C2HC zinc fingers superfamily | -3.10 | 2.82E-02 | 0.73 | 0.23 | Down |
| Csa07g007510 | squamosa promoter-binding 3 | -2.33 | 3.18E-02 | 0.69 | 0.30 | Down |
| Csa13g011890 | flowering locus C | -1.91 | 3.81E-02 | 0.65 | 0.34 | Down |
| Csa04g053240 | AP2-like ethylene-responsive transcription factor SNZ | -3.23 | 9.23E-03 | 0.63 | 0.20 | Down |
| Csa08g053000 | B3 domain-containing transcription factor NGA4-like | -1.93 | 8.81E-03 | 0.63 | 0.32 | Down |
| Csa05g017850 | ABSCISIC ACID-INSENSITIVE 5-like | -2.33 | 3.75E-02 | 0.62 | 0.26 | Down |
| Csa11g031260 | homeobox-leucine zipper HAT1-like | -1.95 | 4.57E-02 | 0.59 | 0.30 | Down |
| Csa01g018970 | ethylene-responsive transcription factor ERF036-like | -2.10 | 5.08E-03 | 0.57 | 0.27 | Down |
| Csa20g066710 | ABSCISIC ACID-INSENSITIVE 5 8 | -4.71 | 1.32E-03 | 0.54 | 0.11 | Down |

| Table S15. Comparative Quantification of transcript levels measured by qRT-PCR and RNA-Seq | | | | |
| --- | --- | --- | --- | --- |
| ID | Gene name | Gene symbol | RNA-Seq  FC | qRT-PCR  FC |
| DEGs in DGAT1#2 line relative to WT | | | | |
| Csa02g065010 | Non-specific lipid transfer 4-like | NSLT-L | 1.51 | 1.75 |
| Csa03g009940 | Glycerol-3-phosphate *sn*-2-acyltransferase 1 | GPAT1 | 1.77 | 0.83 |
| Csa19g001360 | Oleosin 5 | OLE5 | 0.60 | 1.17 |
| Csa11g007400 | 3-Ketoacyl-synthase 18-like | FAE1-L | 0.56 | 1.01 |
| Csa13g016990 | TAG-lipase 2-like | TAGL2-L | 1.77 | 1.427 |
| Csa05g034090 | Acyl CoA thioesterase 13-like | ACOT13-L | 0.51 | 0.812 |
| Csa12g021990 | Cruciferin 3 | CRU3 | 0.244 | 0.203 |
| Csa01g042590 | Acyl-CoA:diacylglycerol acyltransferase 1 | DGAT1 | 1.00 | 1.01 |
| Csa12g028090 | Oleosin 1 | OLE1 | 0.99 | 1.13 |
| Csa02g067560 | glycerol-3-phosphate acyltransferase 9 | GPAT9 | 0.99 | 0.90 |
| Csa06g033410 | lysophosphatidyl acyltransferase 2 | LPAT2 | 1.03 | 1.38 |
| DEGs in GPD1#2 line relative to WT | | | | |
| Csa09g047740 | Glycerol-3-phosphate transporter 1 | GLPT1 | 1.51 | 1.217 |
| Csa01g021830 | Lysophosphatidyl acyltransferase 5 | LPAT5 | 1.95 | 0.64 |
| Csa11g021150 | Glucose-6-phosphate l-epimerase | G6Pe | 1.98 | 2.56 |
| Csa19g024920 | Diacylglycerol kinase 3-like isoform X1 | DAGK | 0.266 | 0.342 |
| Csa16g028550 | 3-Keto acyl-synthase 6 | KCS6 | 0.86 | 0.356 |
| Csa19g023810 | Acyl-activating enzyme 7 | Acylae7 | 0.049 | 0.381 |
| Csa15g015170 | Glycerol-3-phosphate acyltransferase 5 | GPAT5 | 0.125 | 0.69 |
| Csa01g042590 | Acyl-CoA:diacylglycerol acyltransferase 1 | DGAT1 | 0.99 | 0.795 |
| Csa12g028090 | Oleosin 1 | OLE1 | 0.99 | 0.988 |
| Csa02g067560 | glycerol-3-phosphate acyltransferase 9 | GPAT9 | 0.99 | 0.972 |
| Csa06g033410 | lysophosphatidyl acyltransferase 2 | LPAT2 | 1.45 | 1.898 |
| The relative gene expression analysis for the selected candidate genes showing differential regulation in Camelina transgenic lines as compared to non-transgenic WT. Data are the fold changes (FC) in expression measured by using both RNA-Seq and qRT-PCR techniques. DGAT1 #2, the line overexpressing *AtDGAT1*; GPD1 #2, the line overexpressing *ScGPD1*; WT, non-transgenic wildtype. WT data was used as a reference. Red arrows indicate increased levels of gene expression, while green arrows indicate decreased levels of gene expression. | | | | |

| Table S16. List of selected genes used in qRT-PCR analysis. Gene IDs, gene names, gene symbols, primer sequences, and size of amplification products are shown. | | | | |
| --- | --- | --- | --- | --- |
| ID | **Gene name** | **Gene symbol** | **Forward/reverse primers** | **Amplicon size** |
| Csa02g065010 | Non-specific lipid transfer 4-like | NSLT | 5’ GTTGAACGGTATGGCTCAAAC 3’  5’ GGGTAGGGAATGCTAACACC 3’ | 132 bp |
| Csa03g009940 | GPAT1-like | GPAT1 | 5’ TGAGCTGACGGAAGACATTG 3’  5’ GAGACAATAAACCGGCCTAGG 3’ | 133 bp |
| Csa19g001360 | Oleosin 5 | OLE5 | 5’ CTAGCTATGACCGGATTCTTGG 3’  5’ CCTTTGTCCCACGTACTCAG 3’ | 165 bp |
| Csa11g007400 | 3-Ketoacyl-synthase 18-like | KCS-18 | 5’ ATCAAGCACTATTACGTCCCG 3’  5’ CGTTGATCTAGATGCCTCCAC 3’ | 138 bp |
| Csa13g016990 | TAG-lipase 2-like | TAGL2 | 5’ AACTATGGAAGCAGTGATCGG 3’  5’ ACTCCACGTCTTTCACATCG 3’ | 145 bp |
| Csa05g034090 | Acyl CoA thioesterase 13-like | ACT13-L | 5’ TGTGGATGAAGTTGGTGGAG 3’  5’ CCTCTCACGCCTAACAATCTC 3’ | 138 bp |
| Csa12g021990 | Cruciferin 3 | CRU3 | 5’ TTGCAGTACATCAGGCTCAG 3’  5’ ACACATTCTGTCCGTTGTCG 3’ | 151 bp |
| Csa01g042590 | Acyl-CoA:diacylglycerol acyltransferase 1 | DGAT1 | 5’ AAGATACCAAAGACACTCGCC 3’  5’ TGAGCCGAACCTTTCTTGTAG 3’ | 171 bp |
| Csa12g028090 | Oleosin 1 | OLE1 | 5’ GTTGGGAGAGGAAGATAAACCG 3’  5’ GTAGAAGTACTTGGGCCGTAAC 3’ | 195 bp |
| Csa02g067560 | glycerol-3-phosphate acyltransferase 9 | GPAT9 | 5’ AGGGATTGAGTTTGCCGAG 3’  5’ AATCTTGCCAGGATCGACTC 3’ | 156 bp |
| Csa06g033410 | lysophosphatidyl acyltransferase 2 | LPAT2 | 5’ TGACTTTCCTCGACCTTTCTG 3’  5’ TGGCTGGGACAAATGAACG 3’ | 185 bp |
| Csa09g047740 | Glycerol-3-phosphate transporter 1 | G3PT1 | 5’ CTCATGTTCCTAACCGGGATG 3’  5’ GATTATAGCCGTTACCGTAGCC 3’ | 132 bp |
| Csa01g021830 | Lysophosphatidyl acyltransferase 5 | LPAT5 | 5’ AGGTTTCGTCTCCTGCTTG 3’  5’ GGGATTTGGGTTAGTTTGATACG 3’ | 165 bp |
| Csa11g021150 | Glucose-6-phosphate l-epimerase | G6Pe | 5’ CTTTGCCATTGCCTATCACAC 3’  5’ TCAAAGGTTAGTGCATCGCC 3’ | 141 bp |
| Csa19g024920 | Diacylglycerol kinase 3-like isoform X1 | DAGK | 5’ GCGTCGAAGGAGAAATTTGTG 3’  5’ CATAGCTAGACGGAAATACTCAGG 3’ | 162 bp |
| Csa16g028550 | 3-Keto acyl-synthase 6 | KCS6 | 5’ CCTCTTCCTCTTGTCCTTAATCG 3’  5’ CCTCAACGTGTTCTCCTGATAG 3’ | 167 bp |
| Csa19g023810 | Acyl-activating enzyme 7 | Acylae7 | 5’ TGGCGGTGAGAATATAAGCAG 3’  5’ TTCTGGTCTTGCTTCTCGTAAC 3’ | 159 bp |
| Csa15g015170 | Glycerol-3-phosphate acyltransferase 5 | GPAT5 | 5’ CGAGAGGCTGGAAAGGTTTAG 3’  5’ CCGCCAAGATTCTCTGAACATAG 3’ | 156 bp |
| Csa15g026420 | Beta-actin | β-Actin | 5’ ACAATTTCCCGCTCTGCTGTTGTG 3’ 5’ AGGGTTTCTCTCTTCCACATGCCA 3’ | 220 bp |
